# Supplementary material for: Development of conditional-siRNA programmable riboswitch for targeting adverse cardiac remodeling
Source: Mol Ther Nucleic Acids. 2025 Aug 5;36(3):102667. doi: 10.1016/j.omtn.2025.102667 (PMC12375242; doi:10.1016/j.omtn.2025.102667)
Supplement: Document S1. Figures S1–S7 [file mmc1.pdf]

OMTN, Volume *36*

Supplemental information

Development of conditional-siRNA programmable riboswitch for targeting adverse cardiac remodeling

Priyanka Gokulnath, Ane M. Salvador, Caleb Graham, Si-ping Han, Guoping Li, Ramaswamy Kannappan, Christopher Azzam, Michail Spanos, Lisa Scherer, Palaniappan Sethu, John Rossi, William A. Goddard III, and Saumya Das

Supplemental Material

Supplemental Figures – Figure S1-S7

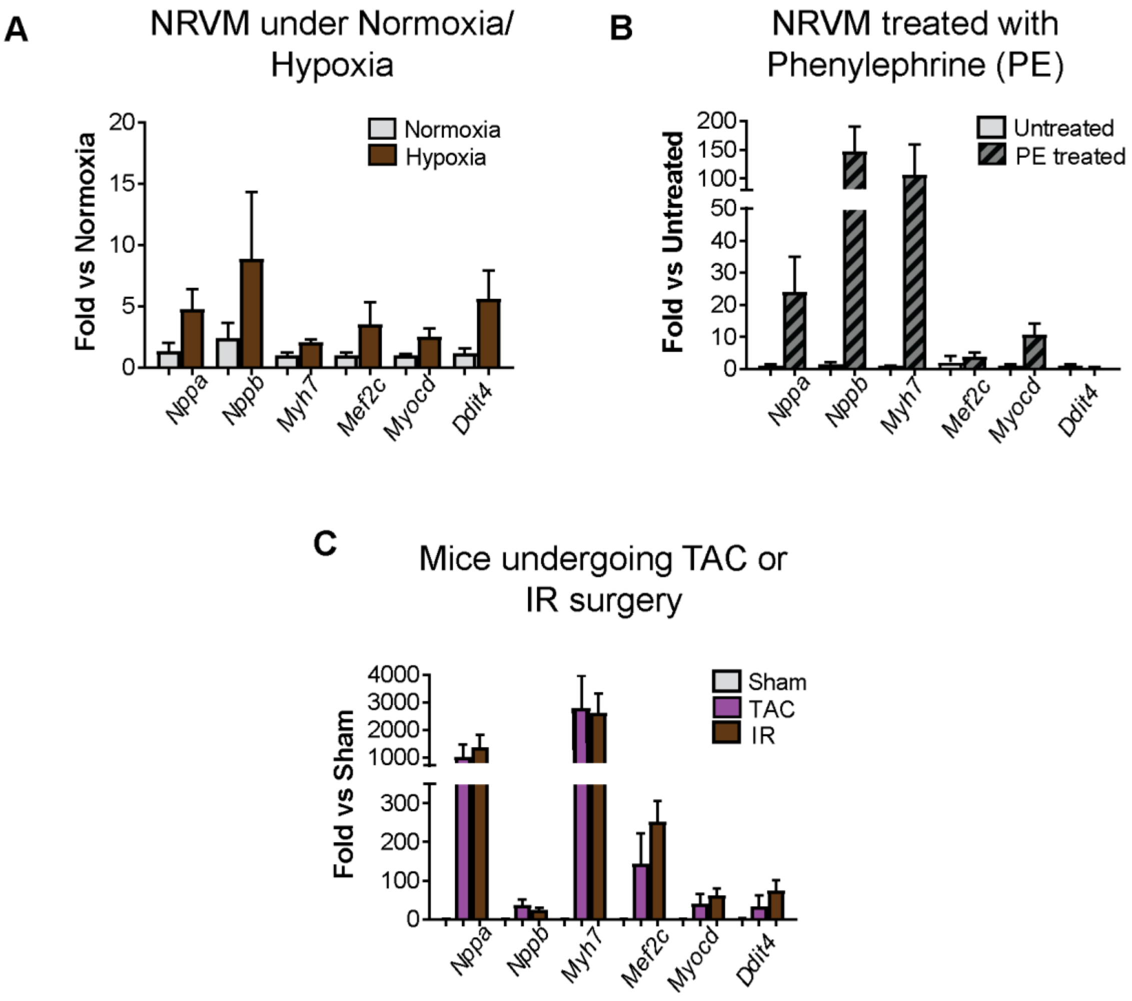

Figure S1. Identification of sensor RNA biomarkers upregulated in response to cardiac stress. Quantification of mRNA levels of cardiomyocyte pathology markers in **A-B**. NRVMs under stress or in mouse hearts **C**. undergoing TAC or IR surgeries. NRVM= Neonatal Rat Ventricular Myocytes, TAC= Transverse Aortic Constriction, IR= Ischemia Reperfusion injury, *Nppa*= Atrial Natriuretic Peptide, *Nppb*= Brain Natriuretic Peptide, *Myh7*= Myosin Heavy Chain beta isoform, *Mef2c*= Myocyte Enhancer factor 2C, *Myocd*= myocardin, *Ddit4*= DNA-damage-inducible transcript 4. Data are shown as Mean (SD) and derived from experiments with n=3.

A Sensor:

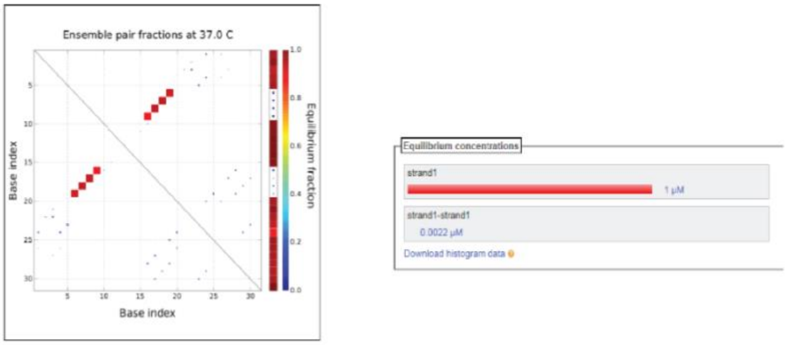

B Sensor + Core:

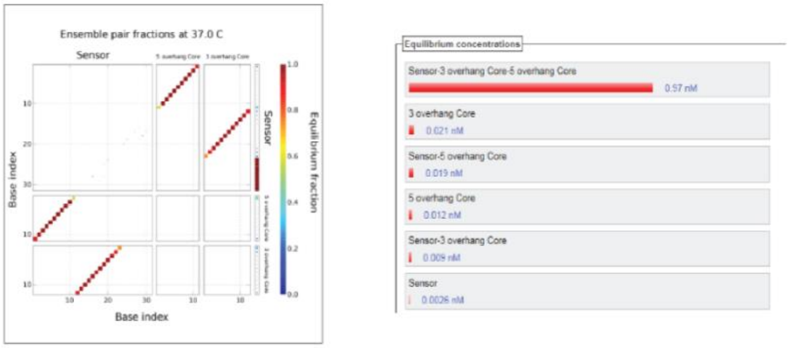

C Guide + Core:

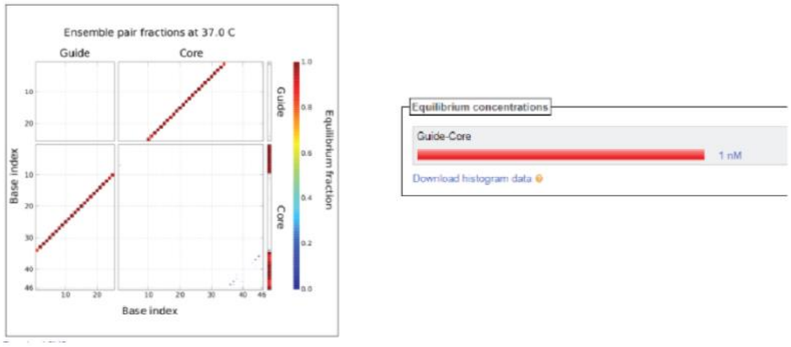

Figure S2. A-C. Nupack software's plots ranking the thermodynamic stability of the complexes formed between the different strands of the *Cond*-siRNA.

A

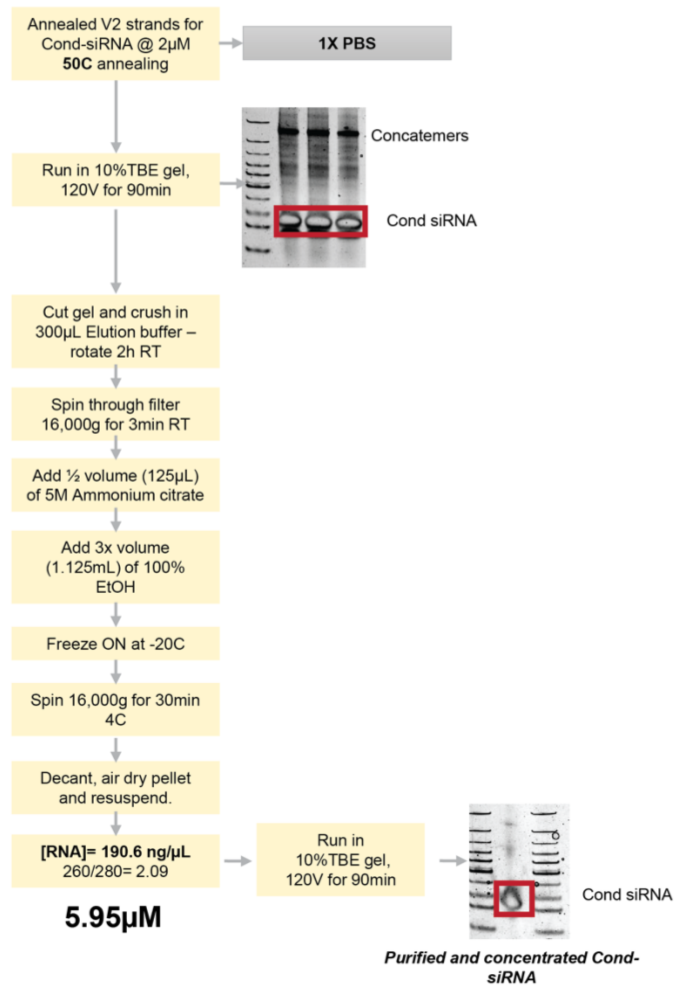

B

Re-annealing :

1. Bring purified construct to 1X PBS adding 10X PBS
2. 25°C to 50°C at 0.1C/s
3. 50°C for 30min
4. Back to 25°C at 0.1C/s

Figure S3. A. Protocol for strand annealing at high concentration, followed by purification to obtain the well-assembled Cond-siRNA and subsequent concentration. **B.** Protocol for re-annealing.

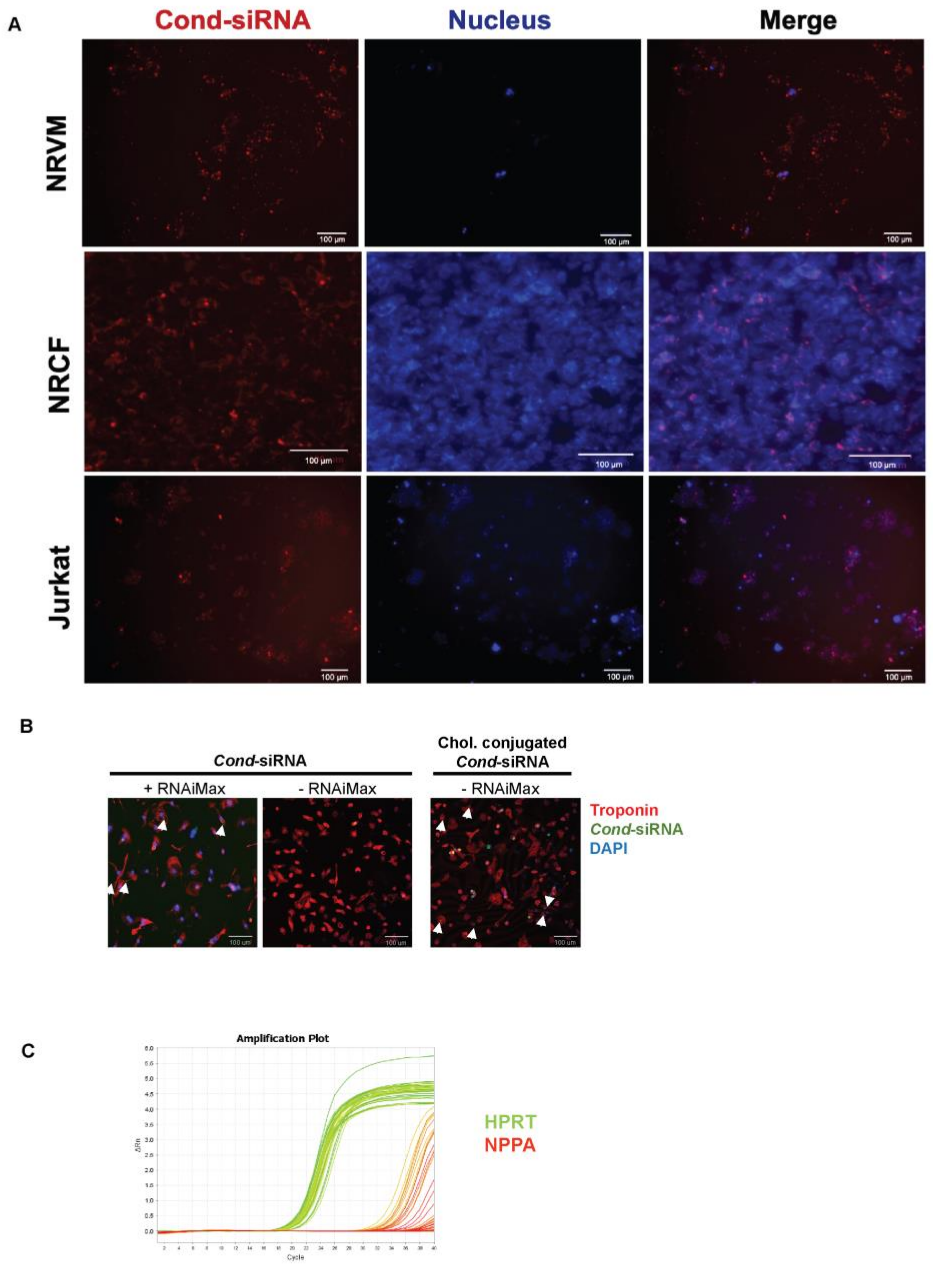

Figure S4. **A**. Representative images showing successful transfection of *Cond*-siRNA (red) in NRVM, NRCFs, and Jurkat cells with DAPi nuclear staining (blue) and Merged images at scale 100 µm. **B**. Representative images of liposomal RNAimax-mediated transfection and direct cholesterol-conjugated Cond-siRNA transfection of NRVMs. *Cond*-siRNA is labeled with FITC, and NRVMs are stained with Troponin T antibody (red) and DAPi nuclear staining (blue). **C.** Representative amplification plot of *Nppa* and *Hprt* (house-keeping gene) in Jurkat cell qRT-PCR.

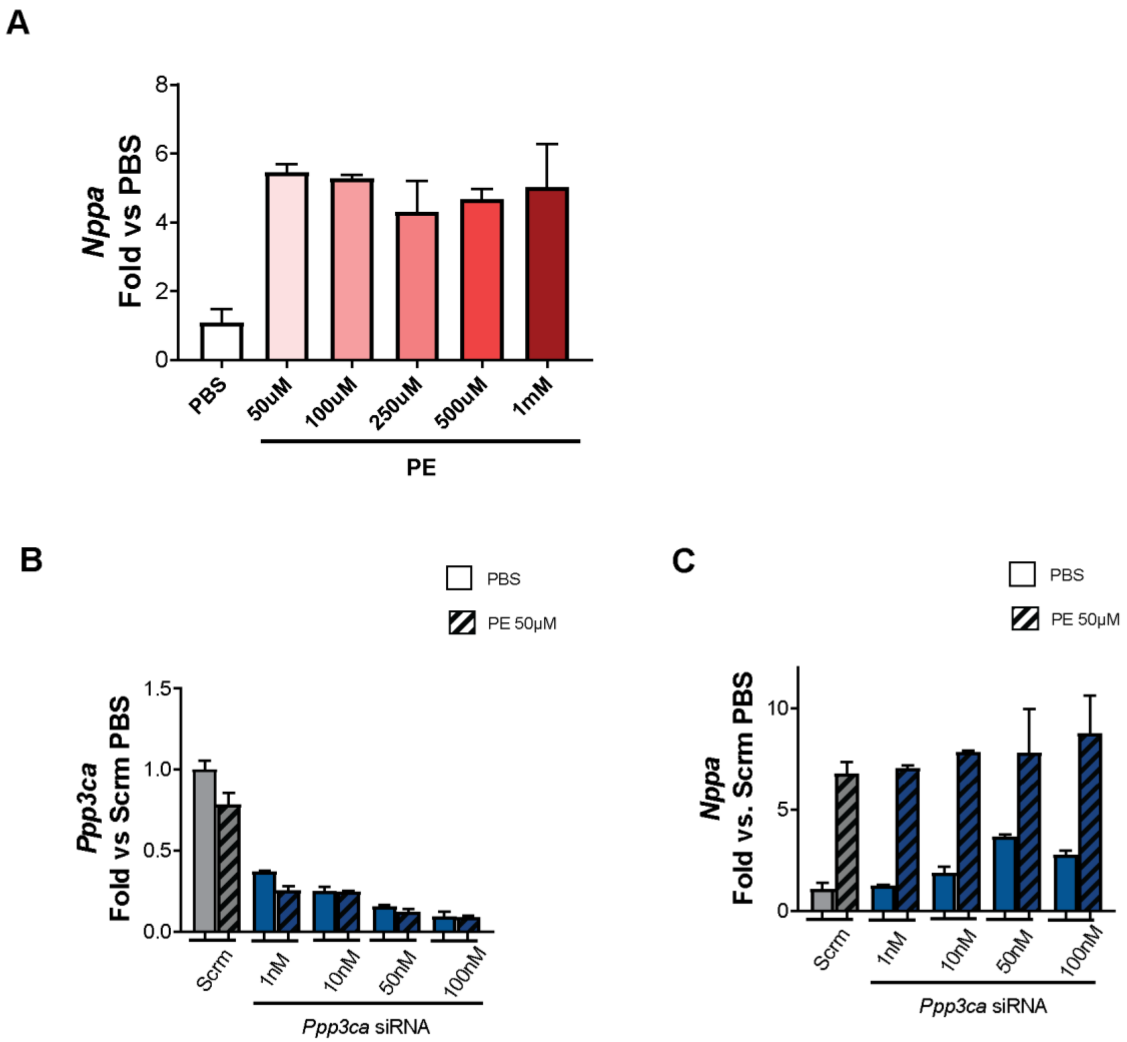

Figure S5. **A.** *Nppa* expression levels in NRVM treated with increasing concentrations of PE for 48 hrs. Data is represented as Fold change normalized to PBS (Fold vs PBS) as mean (SD). **B.** Calcineurin *Ppp3ca* isoform and **C.** *Nppa* mRNA expression in NRVMs transfected with increasing concentrations of commercial siRNA targeting *Ppp3ca*, and treated with 50μM PE. Data is represented as fold change normalized to Scrambled PBS (Fold vs. Scrm PBS) and derived from experiments with n=3 expressed as mean (SD).

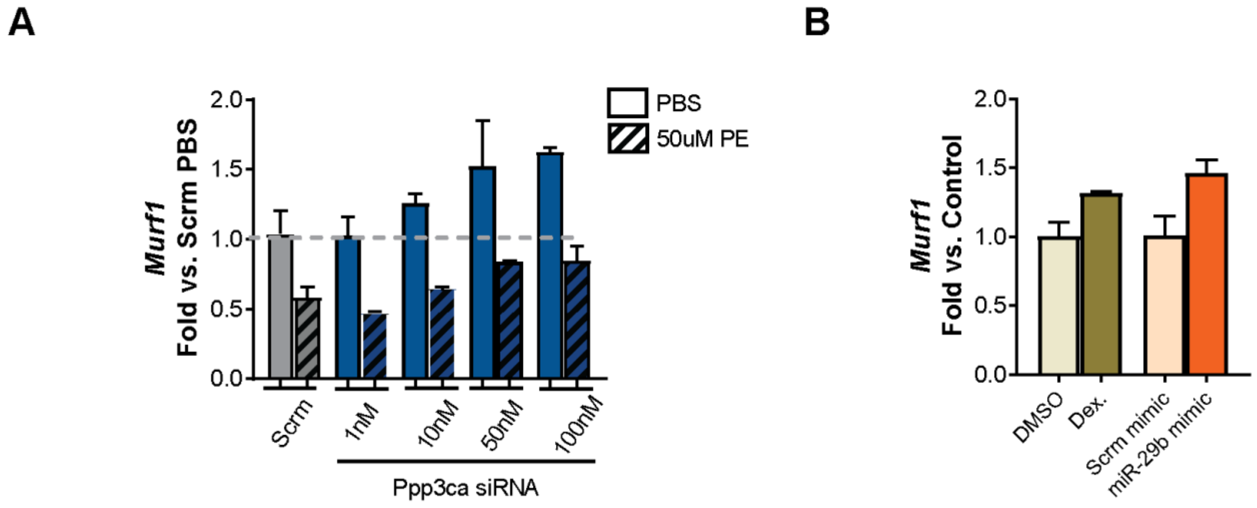

Figure S6. **A.** *Murf1* expression levels in NRVM treated with increasing concentrations of commercial siRNA and PE. Data is represented as fold change normalized to PBS (Fold vs PBS), expressed as mean (SD). **B.** *Murf1* expression levels in NRVM treated with 50µM dexamethasone for 24h or transfected with 50 pmol miR-29b mimic for 48h. Data is represented as fold change normalized to its respective control (Fold vs. control) and derived from experiments with n=3 as mean (SD).

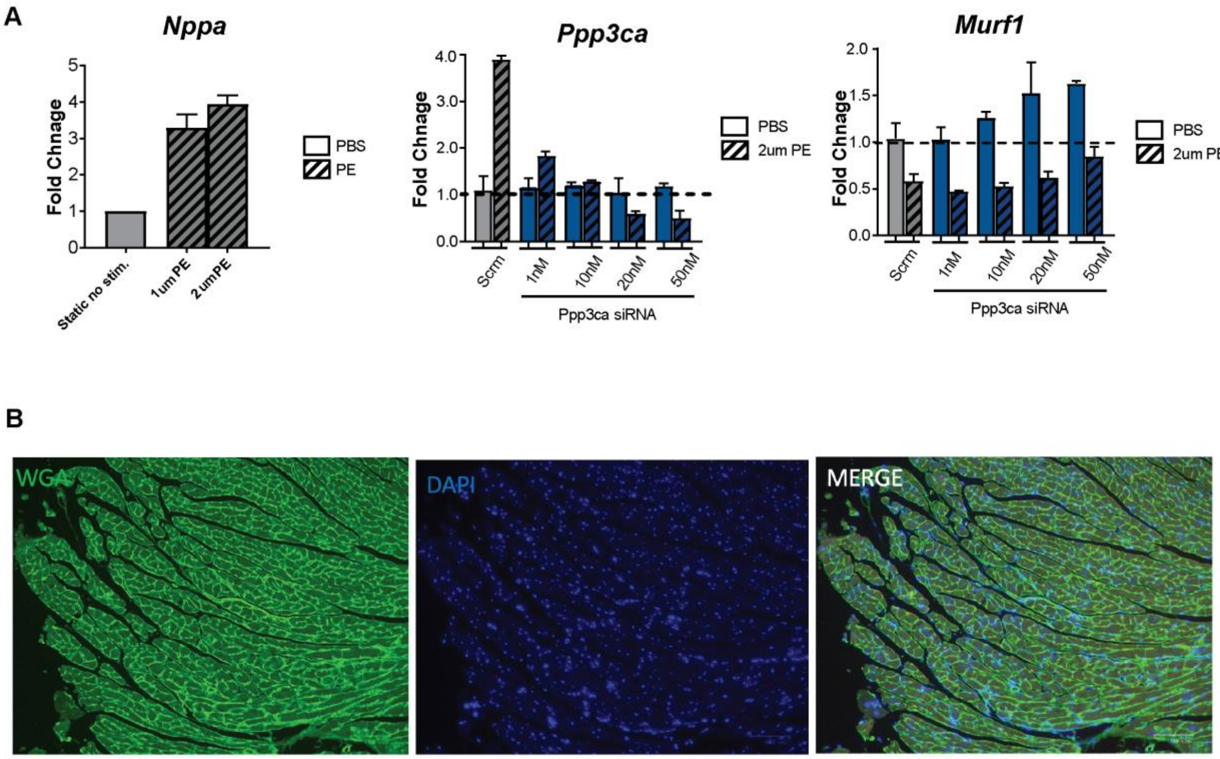

Figure S7. A. Optimal stimulation conditions for inducing *Nppa* upregulation, *Ppp3ca,* and *Murf1* expression in H9c2 fibers on the organ-on-a-chip model administered with different doses of commercial Ppp3ca siRNA (CaN) upon 2 μM PE treatment, evaluated using qRT-PCR. Data is represented as fold change vs its respective control as mean (SD) and derived from experiments with n=3 **B.** Positive immunofluorescence staining of Wheat Germ Agglutinin (WGA, Green) and DAPi nuclear staining (Blue) on heart tissue.

Supplemental Video Material

Video S1. Representative video showing successful transfection of *Cond*-siRNA (red) in beating NRVMs at scale 100 μm, one week after transfection.
