# Supplementary material for: Development of conditional-siRNA programmable riboswitch for targeting adverse cardiac remodeling
Source: Mol Ther Nucleic Acids. 2025 Aug 5;36(3):102667. doi: 10.1016/j.omtn.2025.102667 (PMC12375242; doi:10.1016/j.omtn.2025.102667)
Supplement: Document S2. Article plus supplemental information [file mmc3.pdf]

Molecular Therapy
Nucleic Acids
Original Article

Development of conditional-siRNA programmable riboswitch for targeting adverse cardiac remodeling

Priyanka Gokulnath,[1,5] Ane M. Salvador,[1,5] Caleb Graham,[2] Si-ping Han,[3] Guoping Li,[1] Ramaswamy Kannappan,[2] Christopher Azzam,[1] Michail Spanos,[1] Lisa Scherer,[3] Palaniappan Sethu,[2] John Rossi,[3] William A. Goddard III,[4] and Saumya Das[1]

[1]Cardiovascular Research Center, Massachusetts General Hospital and Harvard Medical School, Boston, MA 02114, USA; [2]University of Alabama at Birmingham, Birmingham, AL 35294, USA; [3]Department of Molecular and Cellular Biology, City of Hope, Duarte, CA 91010, USA; [4]Materials and Process Simulation Center, California Institute of Technology, Pasadena, CA 91125, USA

Heart failure (HF) remains a significant healthcare burden, with an unmet need for novel therapies to target the preceding pathological hypertrophy in HF patients. Here we report the development of novel conditional-siRNA (*Cond*-siRNA) constructs that are selectively activated by disease-specific RNA biomarkers to enable cell-specific inhibition of a target disease-causing RNA. We designed a *Cond*-siRNA that can be activated by *Nppa* mRNA, upregulated specifically in cardiomyocytes (CMs) under pathological stress, to silence the key pro-hypertrophic gene calcineurin (CaN) A-a by the effector small interfering RNA (siRNA). In both neonatal rat ventricular myocytes (NRVMs) and H9c2 CMs, *Cond*-siRNA showed minimal baseline activity but selectively silenced CaN upon *Nppa* mRNA induction by phenylephrine (PE) stress in cell culture models and pressure overload (PO) in a heart-on-a-chip model. In NRVMs, *Cond*-siRNA reduced CaN mRNA only after PE or PO, but not with vehicle, confirming *Nppa*-specific activation. This specificity was further validated as *Cond*-siRNA did not affect CaN in cardiac fibroblasts or T cells lacking *Nppa*. Reduced CaN protein levels and NFATc1 nuclear translocation correlated with decreased NRVM hypertrophy after PE treatment, confirming *Cond*-siRNA's efficacy. This study offers proof-of-concept for *Cond*-siRNA as a targeted therapy to mitigate hypertrophic progression, paving the way for novel HF treatments.

INTRODUCTION

Heart failure (HF) is a leading cause of morbidity and mortality worldwide, affecting over 26 million people worldwide, including 5.7 million in the United States, and represents a major contributor to healthcare expenditure in the US.[1,2] Despite advances in HF treatment to reduce mortality, over 50% of patients die within 5 years from initial diagnosis.[3] The global burden of HF continues to escalate, with a projected 46% increase in HF prevalence by 2030.[3] According to the latest American Heart Association/European Society of Cardiology HF guidelines,[4,5] current therapeutic strategies primarily focus on symptom management and delaying disease progression, yet significant challenges remain in preventing the transition from compensatory hypertrophy to decompensated HF. These alarming trends of the HF epidemic underscore an unmet clinical need for effective therapies that can mitigate HF progression from its preceding pathological hypertrophy in at-risk patients.

Myocardial hypertrophy, characterized by increased cardiomyocyte (CM) size and left ventricular (LV) mass, is a hallmark early compensatory response of the heart to pathological stress.[6,7] Both concentric and eccentric forms of hypertrophy are observed in the initial stages of HF, with concentric hypertrophy predominating in conditions such as hypertension and HF with preserved ejection fraction (HFpEF), and eccentric hypertrophy commonly seen in early HF with reduced ejection fraction (HFrEF) and following myocardial infarction.[8–11] Additionally, hypertrophic cardiomyopathy (HCM) is characterized by pronounced CM hypertrophy and LV mass increase, often with preserved systolic function.[12–15] Importantly, while early-stage HFpEF and HFrEF are marked by increased CM size and LV mass, these features may diminish in end-stage HF as maladaptive remodeling, fibrosis, and myocyte loss predominate.[16–18] Thus, pathological hypertrophy is a critical contributor to HF pathogenesis, with its role varying across disease subtypes and stages.[19,20] Experimental studies in animal models have identified genes that are critical mediators of this adverse cardiac hypertrophy that precedes HF.[21] Notably, genetic and pharmacologic inhibition of key molecules that regulate CM hypertrophy pathways, such as calcineurin (CaN), have shown promise in blocking pathological hypertrophy and its progression to HF.[22–24] However, translating

Received 11 January 2025; accepted 30 July 2025;
https://doi.org/10.1016/j.omtn.2025.102667.

[5]These authors contributed equally

Correspondence: Saumya Das, MD, PhD, Professor of Medicine, Cardiology Division, Cardiovascular Research Center, Massachusetts General Hospital and Harvard Medical School, Boston, MA 02114, USA.
E-mail: sdas@mgh.harvard.edu

© 2025 The Authors.
Published by Elsevier Inc. on behalf of The American Society of Gene and Cell Therapy.
This is an open access article under the CC BY license (http://creativecommons.org/licenses/by/4.0/).

these findings into clinical practice remains challenging due to the off-target effects in other non-cardiac cell types. For example, the critical role of CaN in immune cells suggests that its inhibition in a non-specific manner may lead to adverse side effects.[25]

Recent advances in RNA biology have highlighted its potential for serving as effective therapeutic targets and prompted the development of novel conditional-siRNA (*Cond*-siRNA constructs) or riboswitches.[26] These molecules are specifically activated by signature RNAs that have low baseline expression in normal cellular conditions but are markedly increased in disease states, thereby serving as highly specific markers of pathology in the stressed cell. This enables the cell-specific and state-specific inhibition of a target RNA of the effector site of the *Cond*-siRNA construct. Such a conditional activation confers a high degree of specificity, enabling targeted silencing of disease-associated RNAs and minimizing off-target effects.

In this study, we hypothesize that a riboswitch-based *Cond*-siRNA will ablate pro-hypertrophic signaling, specifically in hypertrophied CMs, and thus ameliorate HF progression. Specifically, we describe an RNA riboswitch that is activated by an mRNA transcript uniquely expressed in hypertrophied CMs, enabling targeted silencing of the CaN gene—a critical mediator of pathological hypertrophy and a novel strategy for HF treatment.

Expanding on our prior study where we demonstrated the feasibility of designing a *Cond*-siRNA to function as prescribed, we report here on the design and experimental validation of a *Cond*-siRNA specifically targeting cardiac hypertrophy in several models. We identify specific molecular sensors for riboswitch activation, evaluate their functionality, and demonstrate their efficacy in validated cardiac hypertrophy cell culture models as well as in a tissue-on-chip model. Our findings reveal that this riboswitch-based *Cond*-siRNA effectively inhibits key pathways involved in adverse cardiac remodeling, achieving robust knockdown activity with minimal off-target effects. These results, in line with our previous proof-of-principle evidence, offer a promising strategy for the targeted treatment of HF, representing a significant advancement in RNA-based therapeutics.

RESULTS

Identification of specific sensors and design of the *Cond*-siRNA targeting cardiac hypertrophy

To identify mRNA transcripts that would serve as sensors suitable to activate the *Cond*-siRNA, we screened several potential sensors by subjecting neonatal rat ventricular myocytes (NRVMs) to two stress models: (1) 24 h of hypoxia (0.2% O_2) followed by 12 h of reoxygenation (Figure S1A), and (2) treatment with the pro-hypertrophic molecule phenylephrine (PE) (Figure S1B). These models mimic pathological stressors that occur in the myocardium during adverse cardiac remodeling. We extended this potential sensor mRNAs' screen to *in vivo* models by examining their expression in heart tissues from mice subjected to either non-ischemic (transverse aortic constriction [TAC]) or ischemic (ischemia-reperfusion [IR]) HF (Figure S1C). Expression levels of candidate mRNAs—Natriuretic peptide A (*Nppa)*, Natriuretic peptide B (*Nppb)*, Myosin Heavy Chain 7 (*Myh7)*, Myosin Heavy Chain 6 (*Myh6)*, Myocyte enhancer factor 2C (*Mef2c)*, Myocardin (*Myocd)*, and DNA damage inducible transcript 4 (*Ddit4)* —were quantified by quantitative reverse-transcription polymerase chain reaction (RT-qPCR). In the hypoxia model, *Nppa* and *Nppb* were markedly upregulated (Figure S1A). These experiments led to the selection of *Nppa*, which encodes for ANP (atrial natriuretic peptide), as the sensor of the *Cond*-siRNAs, based on its low expression in healthy hearts and consistent upregulation in all *in vitro* and *in vivo* cardiac stress conditions.

Synthesis of a *Cond*-siRNA construct based on RNA sensor *Nppa* and target gene *calcineurin*

The design of the *Cond*-siRNA constructs paired the *Nppa* RNA sensor with a target sequence for *CaN A-a* (*Ppp3ca*), using a systematic and iterative protocol to ensure specificity and functionality. The process began with the selection of a validated small interfering RNA (siRNA) sequence targeting *CaN*, which was used as the guide strand. To create a functional 23-base pair Dicer substrate, four GC-rich bases were added to the 5′ end of the guide strand, enhancing its stability and processing efficiency. For the input biomarker strand, the sequence was derived from the 3′ untranslated region (3′ UTR) of *Nppa* mRNA, a region known for its consistent upregulation during cardiac stress (GenBank: NM_012612.2). Computational screening identified potential 31–33 nucleotide antisense segments, with candidates prioritized based on ∼50% GC content and the absence of problematic motifs such as GGGG or MMMM (M = A/U). The reverse complement of each segment was generated as the candidate sensor sequence, which was then validated using NCBI BLAST to exclude unintended matches to the mouse or rat genome.

Using the selected sensor strand, complementary segments of 11 and 12 bases were generated as the putative core strand, designed to interact with the first 23 bases of the sensor strand. Thermodynamic properties, such as binding affinity and secondary structure, were evaluated using Nupack, a nucleic acid ensemble structure prediction package.[27] This analysis confirmed that the sensor strand exhibited minimal secondary structure and predicted strong and accurate binding between the sensor strand and the core strand's 5′ and 3′ overhangs (Figures S2A and S2B). The core strand was then finalized according to the complementary pattern for the *Cond*-siRNA design. For example, the sequences for the guide strand (5′-CG AG UGU UGU UUGGC UU UUCCUG UU-3′), the sensor strand (5′-CUU CACCACCU CUCAGUGGCAAU GCGACCAA-3′), and the core strand (5′-AGGUGGUGAAG CAGGAAAAGCCAAACAACACU CG AUUGCCACUGAG-3′) were systematically aligned to achieve precise duplex formation. Further analysis using Nupack confirmed minimal secondary structure in the core strand overhangs and ensured reliable binding between all components (Figure S2C).

Chemical modifications were added to the *Cond*-siRNA strands to optimize functionality and stability. These modifications included

locked nucleic acid (LNA) substitutions for enhanced target binding affinity and conjugation with cholesterol to improve cellular uptake. An improved version of the initial *Cond*-siRNA design with reduced phosphorothioate modifications of the sensor and an additional LNA modification in the middle toehold has been designated as the first-generation (1st G) construct.[26] Based on this 1st G construct, a second generation (2nd G) construct was altered with additional 2′-O-methyl modifications of the guide strand to increase stability and reduce potential off-target effects. Further refinements were made to improve delivery by conjugating the new sensor with cholesterol (Chol. Conj) and triethylene glycol (Figure 1A). Following synthesis, the *Cond*-siRNA strands were mixed in equimolar concentrations in PBS buffer and thermally annealed using a thermocycler to facilitate precise self-assembly through base-pairing for each of the types of *Cond*-siRNAs (1st G, 2nd G, and Chol. Conj). The assembled *Cond*-siRNAs were then purified via native gel electrophoresis, which confirmed successful assembly, as evidenced by a clean band corresponding to the *Cond*-siRNA construct (Figures 1B and 1C).

This approach represents the successful design of a *Cond*-siRNA targeting *CaN A-a* (*Ppp3ca*), a key gene involved in pathological cardiac hypertrophy.[28] The method combines advanced computational tools, biochemical analysis, and chemical optimization to ensure high specificity, efficient assembly, and effective cellular delivery. This novel strategy lays the groundwork for therapeutic applications targeting adverse cardiac remodeling with precision and minimal off-target effects.

The annealing and purification process for *Cond*-siRNA constructs was optimized to produce well-assembled, concentrated constructs suitable for high-dose transfections. Strand annealing was tested under varying temperatures and salt concentrations to maximize yield while minimizing concatemers and unannealed strands. As shown in Figure S3A, optimal results were achieved using 1× PBS as the annealing buffer, avoiding the high-molecular-weight concatemers observed at low (0.1× PBS) and high (5–10× PBS) salt concentrations. Post-purification, *Cond*-siRNA concentrations were increased from 2 μM to approximately 6 μM using ammonium citrate and ethanol-based methods (Figure S3A). To further ensure proper assembly and conformation, a "re-annealing" step at 50°C was introduced (Figure S3B), resulting in high-quality *Cond*-siRNA constructs free of by-products. This optimized methodology provides well-concentrated *Cond*-siRNA constructs for targeting key mediators of pathological cardiac hypertrophy.

Evaluation of the *in vitro* efficiency of activation of the *Cond*-siRNA construct by the sensor and ability to block its target gene

To assess the efficiency as well as the selective nature of *Cond*-siRNA activation and its ability to block *CaN* expression, we evaluated its uptake, activation, and specificity in *in vitro* models. We conjugated *Cond*-siRNA with Alexa Fluor 546 and transfected them onto NRVMs, neonatal rat cardiac fibroblasts (NRCFs), and Jurkat cells, which showed >90% transfection efficiency in all these cell types (Figure 2A; Video S1; Figure S4A). To increase the robustness of the uptake experiments, NRVMs were also transfected with fluorescein isothiocyanate-labeled *Cond*-siRNA constructs, and their colocalization with CM-specific troponin staining was observed specifically with the cholesterol-conjugated (Chol) *Cond*-siRNA (Figure S4B). While RNAiMax was required for the transfection of unconjugated *Cond*-siRNA, Chol constructs entered cells without the need for transfection reagents, highlighting their enhanced delivery potential.

Having demonstrated the uptake of the *Cond*-siRNA, the activation and siRNA activity efficacy of the *Cond*-siRNA were evaluated in NRVMs under PE-induced stress conditions (Figure S5A) where we saw robust increase in *Nppa* mRNA (the sensor for the *Cond*-siRNAs), as compared to the vehicle buffer (PBS). In the context of PE treatment which increased the sensor *Nppa* mRNA (Figure 2B), NRVM transfection with 1 nM of 1st G, 2nd G, or Chol *Cond*-siRNA construct resulted in a 50% decrease in the expression of CaN (Figure 2C). Importantly, no reduction in *Ppp3ca* expression was observed in PBS-treated cells, further validating the conditional nature of the constructs, which respond specifically to PE-induced *Nppa* upregulation, with minimal silencing of the target *Ppp3ca* in the absence of the disease biomarker *Nppa* (Figure 2C). This was in contrast to the commercial siRNA targeting *Ppp3ca*, that resulted in silencing of the target in both PBS- and PE-treated cells (Figures 2C–2E). Consistent with prior studies, we did not see a significant increase in *Ppp3ca* mRNA with PE treatment in the control scrambled siRNA group.[28,29] A dose-response was noted with transfection of NRVMs with higher doses (10 and 30 nM) of the 1st G *Cond*-siRNA (Figures 2D and 2E) leading to further reductions in CaN silencing, achieving a 70%–80% decrease in expression. The degree of CaN silencing achieved with the *Cond*-siRNA was similar to that observed when transfecting commercial CaN targeting siRNA into NRVMs (Figures S5B and S5C showing *Ppp3ca* and *Nppa*, respectively).

To demonstrate the CM-specific activation and siRNA activity of the *Cond*-siRNA, neonatal rat cardiac fibroblasts were utilized to quantify the expression of CaN after treatment with 1 nM of the different variants of the *Cond*-siRNA in the presence of PE (Figure 2F). As expected, the *Cond*-siRNA did not reduce CaN expression in the presence of PE, reaffirming the cell-specific activation and siRNA activity of the *Cond*-siRNA, restrained to *Nppa* expressing NRVMs. Furthermore, considering the important function of CaN in T cell activation and effector function, the *Cond*-siRNA activity was determined in immortalized human Jurkat T cells (Figure 2G; Figures S4A and S4C). Given that the *Cond*-siRNA gets activated only in the presence of *Nppa* mRNA, the construct should not get activated in T cells (see *Nppa* amplification plot in Figure S4C), and therefore, it would not alter T cell activation and immune competence. While electroporation of Jurkat cells with a commercial siRNA targeting CaN led to ∼50% decrease in CaN expression, treatment with 1 nM *Cond*-siRNA and Chol *Cond*-siRNA did not result in significant changes in CaN expression in the presence or absence of PE, further validating CM *Nppa* specific activation of the constructs and therefore

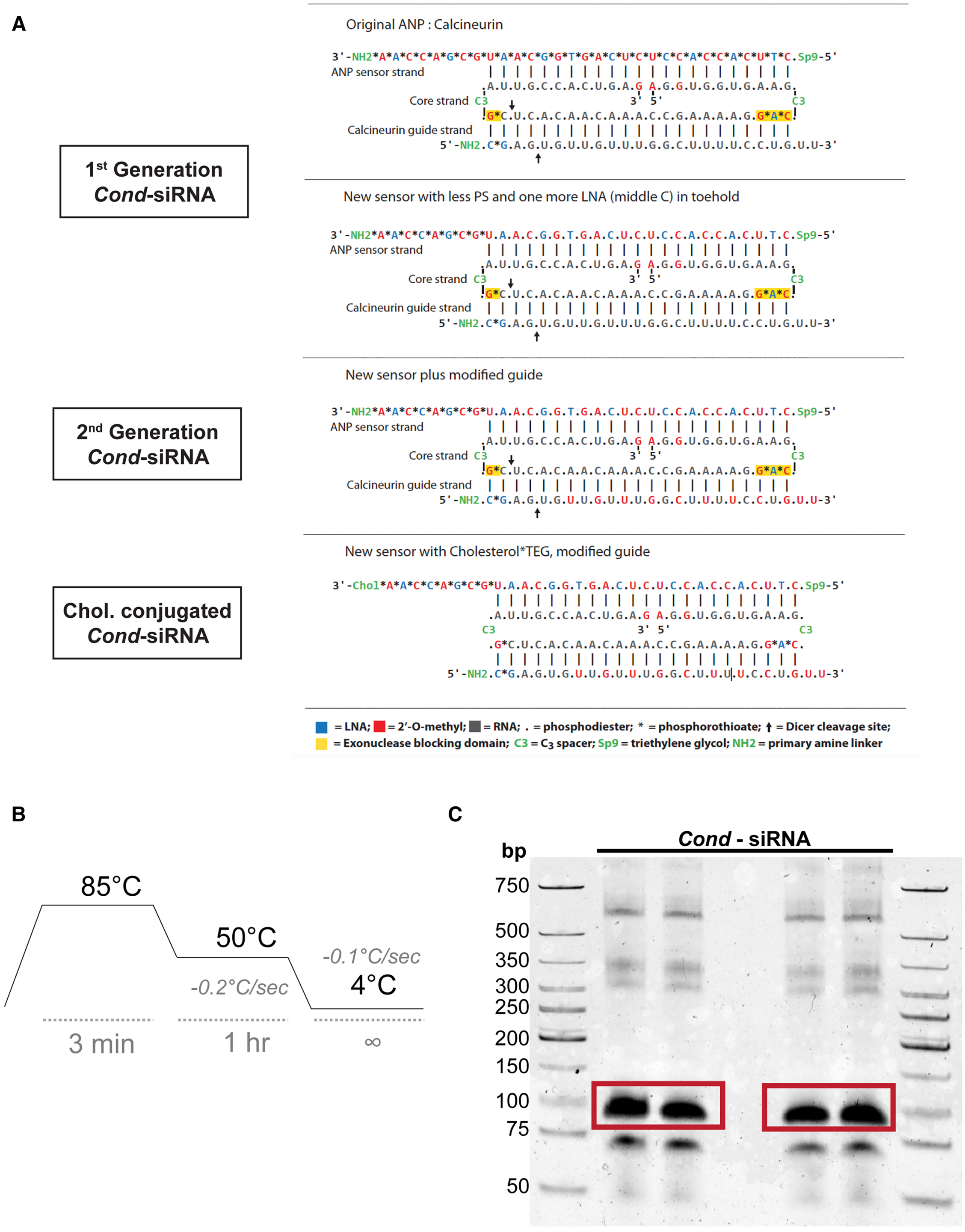

Figure 1. Synthesis of a *Cond*-siRNA construct based on RNA sensor *Nppa* and target gene calcineurin

(A) Conditional-siRNA (*Cond*-siRNA) constructs' designs, based on *Nppa* mRNA sensor and the target gene calcineurin. Each of the different constructs bears different chemical modifications, indicated below in a color-coded format, which cf. thermodynamic stability or cell-penetrating capacity. (B) Thermocycler program used to induce base-pairing driven self-assembly of the different strands combined in an equimolecular manner to generate the *Cond*-siRNA via thermal annealing. (C) Assembled 1st G *Cond*-siRNA constructs run in a 10% TBE appear as a clean band (highlighted in a red square). Higher molecular weight products correspond to concatemers of the different RNA strands, and smaller molecular weight products correspond to strands that did not assemble in the *Cond*-siRNA constructs.

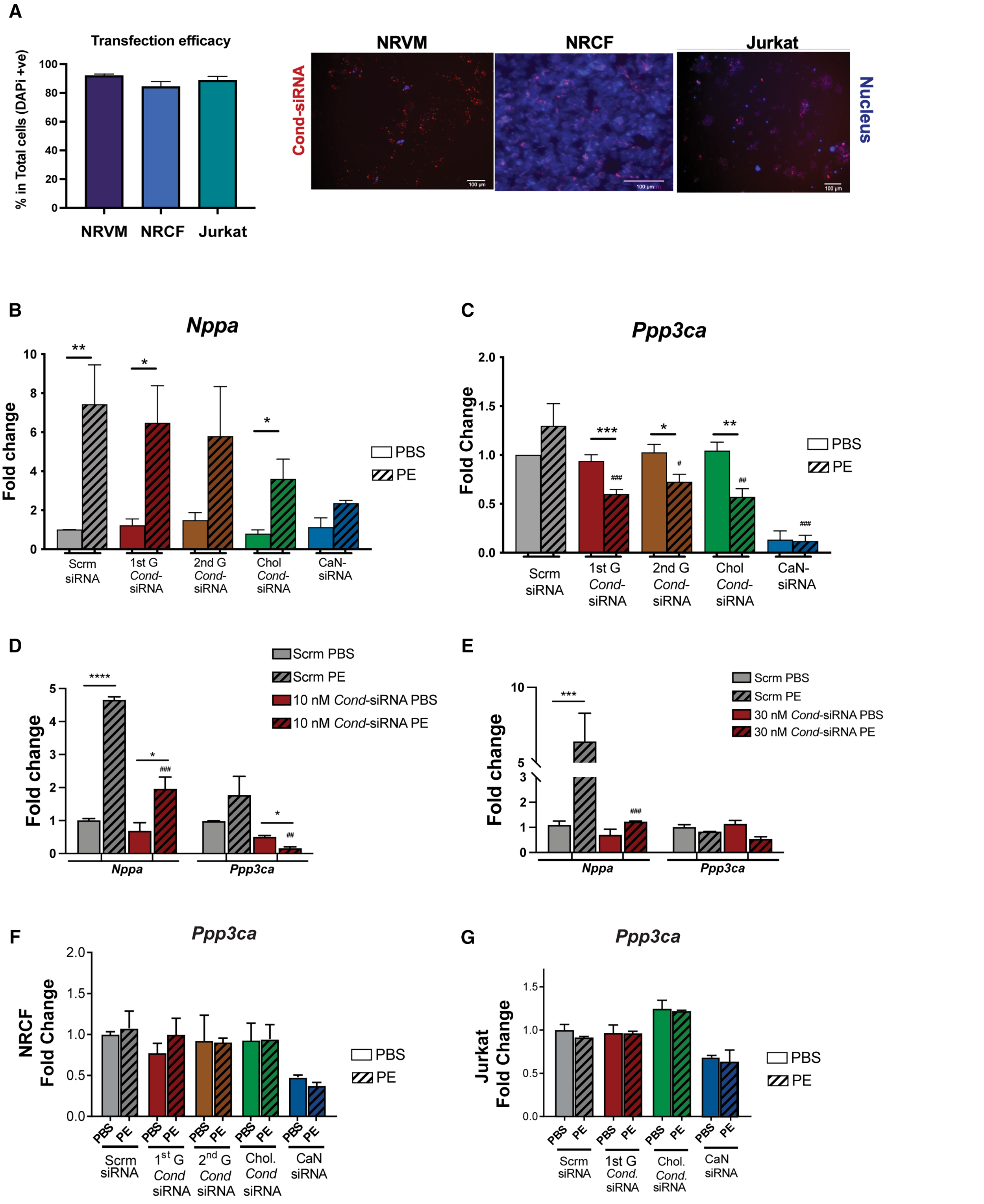

Figure 2. Cardiomyocyte-specific activation and siRNA activity of *Cond*-siRNA

(A) Successful transfection of *Cond*-siRNA (red) with DAPI nuclear staining (blue) into NRVM, NRCFs, and Jurkat cells with representative images with scale bars of 100 μm. (B–E) Atrial natriuretic peptide (*Nppa*) and calcineurin (*Ppp3ca*) mRNA expression in NRVMs treated with either 1 nM (B and C) of the different *Cond*-siRNA constructs and

(legend continued on next page)

suggesting a lack of off-target immunosuppressive effects when using the *Cond*-siRNA *in vivo*.

Finally, concordant to the RNA expression, treatment with *Cond*-siRNA in the presence of PE resulted in a significant reduction in CaN (PPP3CA) protein levels across all *Cond*-siRNA constructs compared to scrambled siRNA, when normalized to GAPDH. This confirms that *Cond*-siRNA decreases expression of CaN A-alpha protein in the setting of PE stimulus (concordant with changes in the mRNA for *Ppp3ca*). Interestingly, we did see a modest increase in PPP3CA protein in the control scrambled group in the presence of PE. As expected, CaN levels remained unchanged in PBS-treated cells, consistent with the conditional activation mechanism of the siRNA.

Treatment of NRVMs with the *Cond*-siRNA leads to inhibition of NFATc1's nuclear translocation and pathological hypertrophy without promoting myocyte atrophy

Having demonstrated the siRNA activity of the *Cond*-siRNA in NRVMs in the presence of PE-induced upregulation of *Nppa*, we next sought to characterize the effects of the *Cond*-siRNAs on CaN and its downstream signaling and the expression of other pathological cardiac hypertrophy markers under baseline (PBS) or PE treatment.[30,31] In these experiments, we determined if each of the *Cond*-siRNA constructs could mitigate the PE-induced changes noted in the control scrambled siRNA group. We first checked the expression of the CaN protein, which decreased in all the different *Cond*-siRNA treatments with respect to the scrambled control (Figures 3A and 3B). Additionally, we compared the expression levels of these markers in the presence of PE across the different treatment groups. We noted that PE treatment resulted in an increase in PPP3CA (Figures 3A and 3B), nuclear factor of activated T-cells c1 (NFATc1, Figures 3C and 3D), increased phosphorylation of extracellular signal-regulated kinase 1/2 (ERK1/2) (Figures 3E and 3F), a trend toward increased myosin heavy chain 7 (MYH7) protein (Figures 3E and 3G) and an increase in the *Myh7/Myh6* mRNA ratio that is characteristic of pathological hypertrophy (Figure 3H). Notably, this increase with PE was abolished or mitigated in the cells treated with the *Cond*-siRNAs (Figures 3A–3H), especially for 1st G *Cond*-siRNA and Chol *Cond*-siRNA. When compared to the PE-treated scrambled siRNA, the levels of these markers were all decreased in the *Cond*-siRNA groups, consistent with their state-specific anti-hypertrophic effect. Unexpectedly, the 2nd generation *Cond*-siRNA also appeared to decrease NFATc1 levels in the PBS group (independent of its effect on PPP3CA), suggesting an off-target effect or a cellular stress response to this chemical entity.

To evaluate the potent anti-hypertrophic effects of the *Cond*-siRNA, we verified if muscle atrophy pathways were upregulated, which would be deleterious as a treatment side effect. To test this, mRNA levels of muscle RING-finger protein 1 (*Murf1)* were quantified, finding that no significant upregulation was observed at 1 and 10 nM concentrations (Figures 3I and 3J). This mimics the expression of this atrophy marker in NRVMs treated with commercial siRNAs targeting CaN (Figure S6A). Interestingly, further silencing of CaN using higher concentrations of *Cond*-siRNA (30 nM) or commercial siRNA (50 and 100 nM, Figure S6A) led to an increase in the expression of atrophy marker *Murf1*. As a positive control of muscle atrophy, dexamethasone and miRNA-29b were utilized,[32] where their treatment or transfection to NRVM led to an upregulation of the atrophy marker *Murf1* (Figure S6B). Therefore, the upregulation of atrophic genes when severely silencing CaN expression points toward a therapeutic window to decrease CaN levels but without inducing NRVM atrophy.

CaN silencing mediated by the *Cond*-siRNA attenuates PE-induced CM hypertrophy. To assess the corresponding phenotypical effect of CaN silencing by the *Cond*-siRNA on the cells, the NRVM area was quantified upon transfection of the different generations of the *Cond*-siRNA, in the presence or absence of PE. Staining of NRVMs with troponin enabled the tracing and measurement of cell area and showed that the reduced CaN expression in cells treated with the *Cond*-siRNA exposed to PE reduced cellular hypertrophy phenotype, presenting significantly lower NRVM area after PE treatment, as compared to scramble siRNA-treated NRVMs (Figure 4), concordant with the changes seen in the pro-hypertrophic signaling pathway.

Evaluation of *Cond*-siRNA in a heart-on-chip model under pressure-overload condition

A preclinical heart-on-chip (HOC) model was successfully developed previously to simulate HF conditions induced by pressure overload (PO).[33] Engineered tissues fabricated with polydimethylsiloxane (PDMS) at a 10:1 base-to-crosslinker ratio provided a robust framework for housing H9c2 (rat CM) tissues in a 4 mL media chamber.

commercial siRNA, (D) 10 nM, and (E) 30 nM of 1st G *Cond*-siRNA. Twenty-four hours post-isolation, NRVMs were transfected with the *Cond*-siRNA; after 24 h, NRVMs were treated with 50 μM PE for 48 h, and RNA expression was determined 72 h post-transfection. (F) Calcineurin (*Ppp3ca*) mRNA expression in NRCFs treated with 1 nM of the different *Cond*-siRNA constructs or the commercial calcineurin siRNA. One-week post-isolation, NRCFs were transfected with the *Cond*-siRNA; after 24 h, NRCFs were treated with 50 μM PE for 48 h, and RNA expression was determined 72 h post-transfection. Data are shown as fold change normalized to the scrambled PBS group with housekeeping control (*Actb*) using the ddct method. $n = 3$ with data represented as mean (SD). (G) Calcineurin (*Ppp3ca*) mRNA expression in Jurkat T cells transfected with 1 nM of commercial siRNA (CaN siRNA) or *Cond*-siRNA constructs (1st G and cholesterol-conjugated *Cond*-siRNA). Twenty-four hours after treatment with the *Cond*-siRNA, cells were treated with 50 μM PE for 48 h, and RNA expression was determined 72 h post-treatment. All data are derived from experiments with $n = 4$–9. Data are shown as fold change normalized to the scrambled PBS group with the house-keeping gene (*Actb*) using the ddct method. Unpaired t test was performed between PBS and PE groups with significance indicated as *$p \leq 0.05$, **$p \leq 0.01$, and ***$p \leq 0.001$; and between scrambled PE vs. other PE groups using ANOVA with significance indicated as #$p \leq 0.05$, ##$p \leq 0.01$, and ###$p \leq 0.001$.

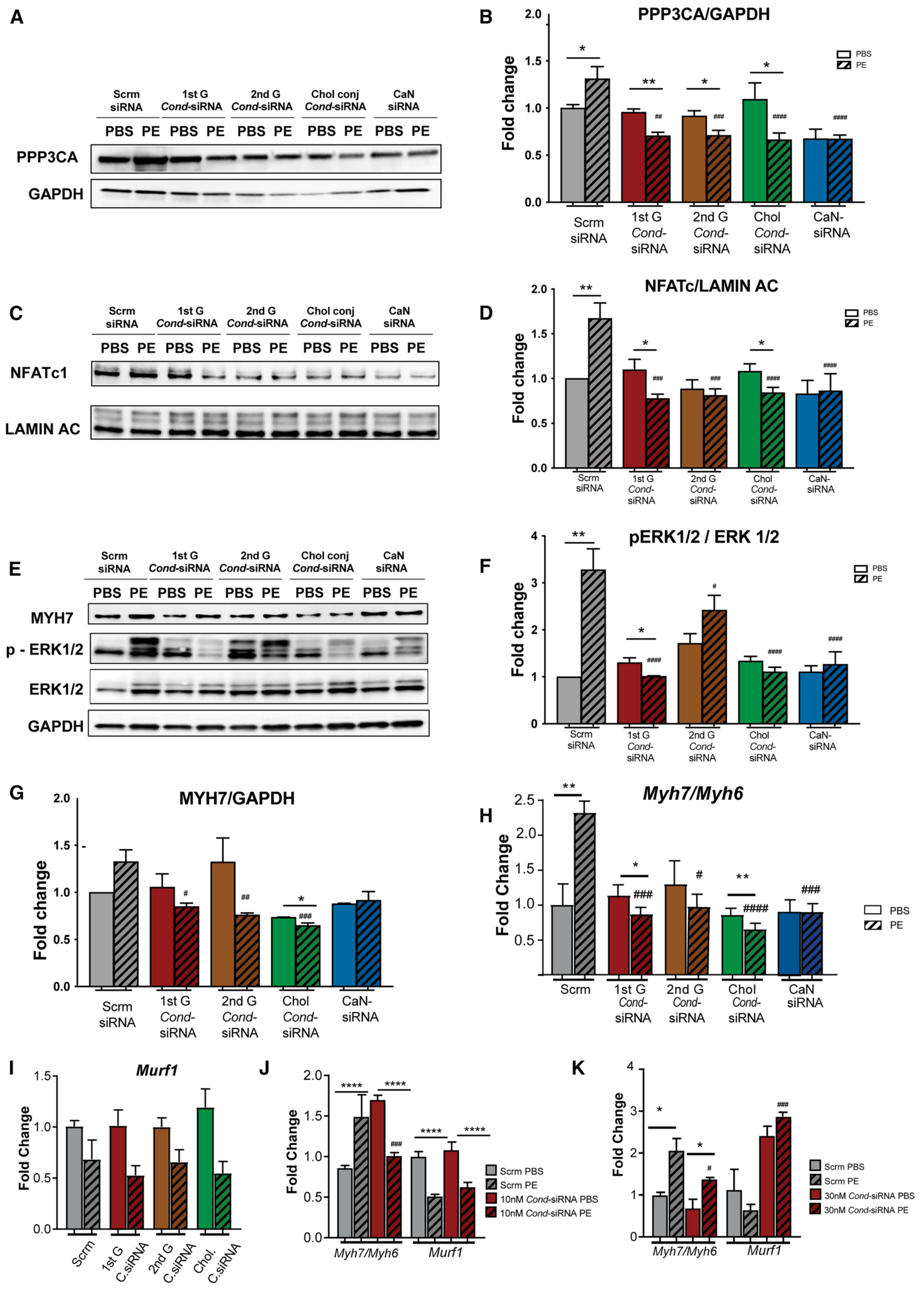

(legend on next page)

H9c2 cells were cultured, transfected, and incorporated into fibrin-based fibers, which were subjected to either static conditions (ST) or PO (200/10 mmHg) optimized with PE treatment as a positive control (Figure S7). The *Cond*-siRNA conditions were determined based on a dose-response experiment, and preliminary results suggested the requirement of a concentration of 20 nM of the commercial CaN siRNA without activating the *Murf1* pathway (Figure S7). Three transfection groups consisting of 20 nM of scrambled siRNA (Scr), commercial CaN siRNA (CaN siRNA), and *Cond*-siRNA (1st G) were tested, allowing a comparative analysis of targeted siRNA interventions (Figure 5A). We focused on the 1st G *Cond*-siRNA, given the concerns about off-target effects on the hypertrophic signaling pathways seen in the 2nd Gen *Cond*-siRNA discussed previously.

Under PO, the expression of *Nppa* was elevated across all treatment groups, indicating a general response to pathological stress (Figure 5B). However, the upregulation of *Nppb*, a marker of pathological hypertrophy, observed in the Scr_PO group was markedly diminished in the *Cond*-siRNA and siRNA groups, suggesting amelioration of the pathological hypertrophy stress response induced by PO (Figure 5C). The expression of CaN isoforms *Ppp3ca* and protein phosphatase 3 catalytic subunit beta (*Ppp3cb)* was analyzed to assess the effectiveness of siRNA-mediated knockdown. In the Scr_PO group, both isoforms were significantly upregulated under PO. Treatment with *Cond*-siRNA selectively reduced the expression of *Ppp3ca* under PO conditions while maintaining basal levels under ST, demonstrating its conditional activation (Figure 5D). However, *Cond*-siRNA had no significant effect on *Ppp3cb* expression in the ST groups, but reversed its increase in the Scr_PO group (Figure 5E), likely secondary to its anti-hypertrophic effect. In contrast, as expected, CaN siRNA silenced the *Ppp3ca* isoform under all conditions, irrespective of stress, underscoring the stress-dependent activity of the *Cond*-siRNA for conditional knockdown over the CaN siRNA. The *Myh7/Myh6* ratio, a well-established marker of pathological cardiac remodeling, was significantly elevated in the Scr_PO group, reflecting hypertrophic changes (Figure 5F). This increase was prevented in the *Cond*-siRNA_PO group, indicating its efficacy in mitigating pathological remodeling and maintaining a physiological phenotype.

Morphological changes were assessed using wheat germ agglutinin (WGA) and 4′,6-diamidino-2-phenylindole (DAPI) nuclear staining (Figure 5G), with positive staining on mouse heart tissue indicated in Figure S7B. Under ST, H9c2 fibers appeared compact and uniform. In contrast, exposure to PO resulted in elongated fibers with wider diameters, reflecting hypertrophic remodeling in the Scr_PO group. Cells in the *Cond*-siRNA_PO group, however, retained a morphology smaller than the Scr_PO group, indicating a protective effect against PO-induced hypertrophy. Quantification of cellular diameters (Figure 5H) confirmed these observations, with significantly smaller diameters in the *Cond*-siRNA_PO group compared to Scr_PO ($p < 0.0001$). These findings validate the conditional and stress-specific activation of *Cond*-siRNA, which selectively silences *Ppp3ca* under pathological conditions, effectively mitigating hypertrophic remodeling under PO conditions, highlighting its potential as a targeted therapeutic strategy for HF.

DISCUSSION

HF often progresses silently, with patients experiencing subclinical cardiac remodeling long before the onset of symptoms. Pathological cardiac hypertrophy is an early hallmark of adverse cardiac remodeling that precedes overt HF.[16,34] Early intervention, particularly through targeted therapies, is crucial for preventing irreversible damage and improving long-term outcomes. Recent guidelines highlight the importance of initiating preventive measures in high-risk populations, such as those with hypertension, diabetes, or a family history of HF.[4,5] The findings of this study demonstrate the potential of a novel *Cond*-siRNA construct in selectively targeting and silencing CaN, a key driver of pathological cardiac hypertrophy.

Different approaches have been employed to target cardiac hypertrophy, focusing on key signaling pathways and transcriptional regulators that drive maladaptive remodeling. Recent advancements in RNA-based therapies have significantly broadened the therapeutic landscape for cardiac remodeling.[35–37] siRNAs have shown exceptional potential in precisely targeting molecular pathways driving hypertrophy. For example, siRNA-mediated knockdown of mutant *Myh6* transcripts in mouse models has successfully reduced HCM, demonstrating the efficacy of gene-specific silencing.[38]

Non-coding RNAs, including microRNAs (miRNAs), long non-coding RNAs (lncRNAs), and circular RNAs, are emerging as critical regulators and therapeutic targets for various diseases.[39] miR-30d, for instance, regulates cardiac remodeling through both intracellular

Figure 3. Validation of proteomic and transcriptomic targets on abrogation of pathological hypertrophy by the *Cond*-siRNA without promoting myocyte atrophy

(A–G) Quantification of protein levels of (A and B). Calcineurin (PPP3CA) in the cytoplasm with GAPDH loading control (C and D). NFATc1 in the nucleus with LAMIN A/C as loading control (E–G). *p*-ERK1/2/ERK1/2 (E and F) and MYH7with GAPDH loading control (E and G) in NRVMs treated with 1 nM of the commercial siRNA (CaN siRNA) or the different *Cond*-siRNA constructs. Twenty-four hours post-isolation, NRVMs were transfected with the *Cond*-siRNA; after 24 h, NRVMs were treated with 50 μM PE for 48 h, and protein expression was determined 72 h post-transfection. Data are shown as fold change normalized to the scrambled PBS group. Unpaired t test was performed between PBS and PE groups, with significance indicated as $*p \leq 0.05$, $**p \leq 0.01$, and $***p \leq 0.001$; and between scrambled PE vs. other PE groups using ANOVA with significance indicated as $\#p \leq 0.05$, $\#\#p \leq 0.01$, and $\#\#\#p \leq 0.001$. (H–J) Quantification of mRNA levels of *Myh7/Myh6 ratio* and atrophy marker *Murf1* in NRVMs treated with 1 nM of different *Cond*-siRNA (H and I), and 10 nM (J), and 30 nM (K) of the 1st generation *Cond*-siRNA, treated with 50 μM PE for 48 h. Data are represented as fold change normalized to the scrambled PBS group with housekeeping control (*Actb*) using the ddct method. All data are derived from experiments with $n = 4$. Statistics used are unpaired t test performed between PBS and PE groups, with significance indicated as $*p \leq 0.05$, $**p \leq 0.01$, and $***p \leq 0.001$; and between scrambled PE vs. other PE groups using ANOVA with significance indicated as $\#p \leq 0.05$, $\#\#p \leq 0.01$, and $\#\#\#p \leq 0.001$.

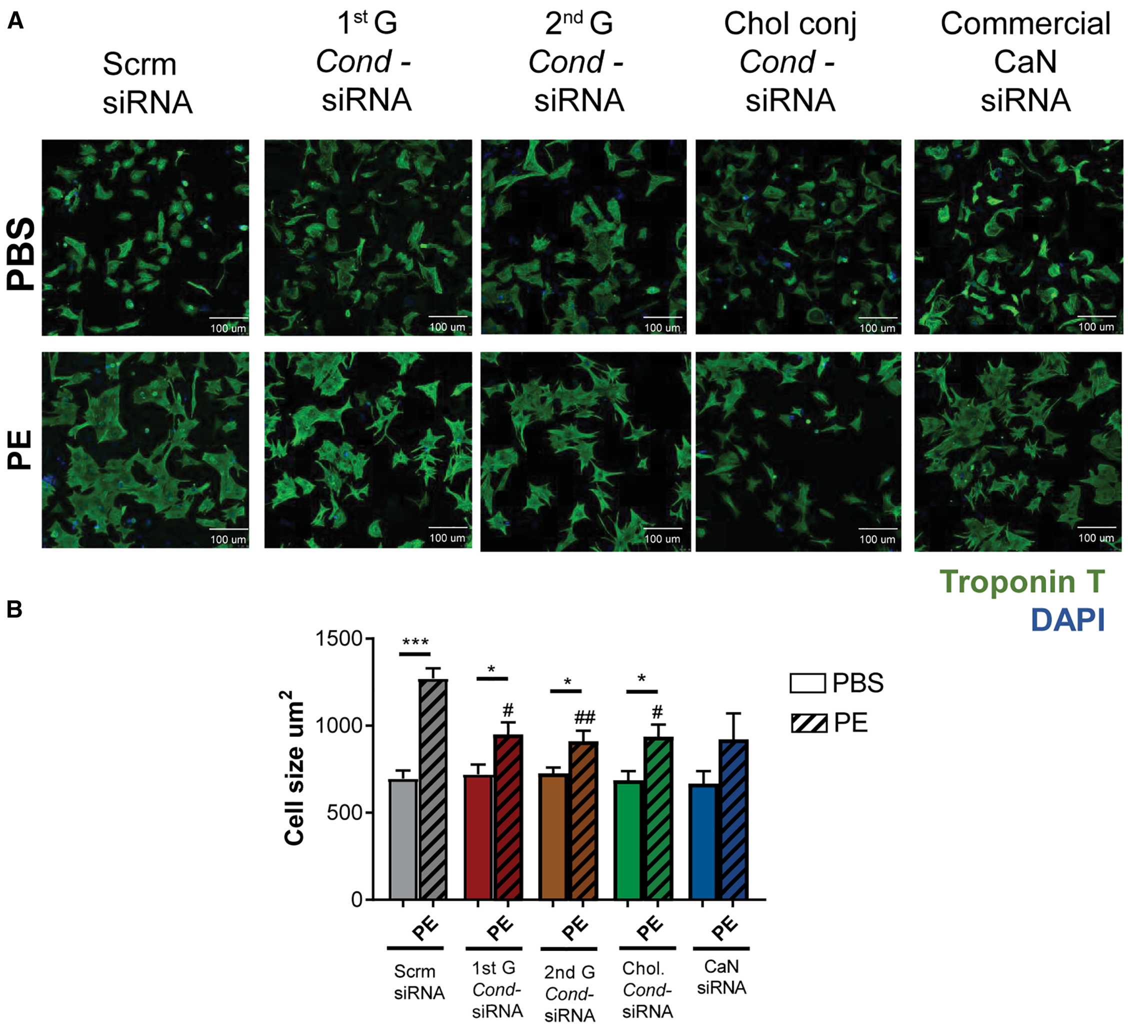

Figure 4. Effect of *Cond*-siRNA on NRVMs

(A) Representative images and (B) Quantification of neonatal rat ventricular myocyte (NRVM) area as a marker of cellular hypertrophy in NRVMs treated with 1 nM of the different *Cond*-siRNA constructs or the commercial calcineurin (CaN) siRNA 24 h post-isolation. NRVMs were transfected with the *Cond*-siRNA; after 24 h, NRVMs were treated with 50 μM PE for 48 h, and cellular area was measured 72 h post-transfection, having stained the cells for Troponin T (green) and DAPI nuclear stain (blue), represented with a scale bar of 100 μm. All data are derived from experiments with $n = 5$ expressed as mean (SD). Unpaired t test was performed between PBS and PE groups and between scrambled PE vs. other PE groups using ANOVA with significance indicated as #$p \leq 0.05$, ##$p \leq 0.01$, and ###$p \leq 0.001$.

signaling and extracellular vesicle-mediated communication. Studies have highlighted its ability to mitigate hypertrophy and fibrosis by modulating critical signaling pathways.[40,41] Antagonizing miR-208a has shown efficacy in reducing hypertrophy by targeting MYH7 expression.[42] Among the molecular regulators of remodeling, CaN remains a key target due to its role in hypertrophic signaling through the activation of NFAT transcription factors. Its inhibition prevents pathological remodeling and has been validated as an effective therapeutic strategy in preclinical models.[43,44]

Additionally, small molecule inhibitors of CaN, have demonstrated improved HF outcomes and reduced hypertrophy.[45] These prior data guided our choice of CaN as an initial target in mitigating cardiac hypertrophy. However, CaN's ubiquitous expression across tissues poses a significant challenge, as its inhibition can lead to widespread off-target effects, limiting its clinical application. Our strategy provides a promising alternative by utilizing *Cond*-siRNA, which addresses these limitations through a disease-specific activation mechanism. By leveraging *Nppa*, a robust and specific biomarker activated exclusively under pathological conditions, this approach ensures precise therapeutic activation, silencing CaN selectively in disease states while sparing normal physiological processes.

As designed, the *Cond*-siRNA construct is only functionally activated in cells that express the relevant trigger *Nppa* mRNA, such as in CMs subjected to hypertrophy stimuli, which explains the selective

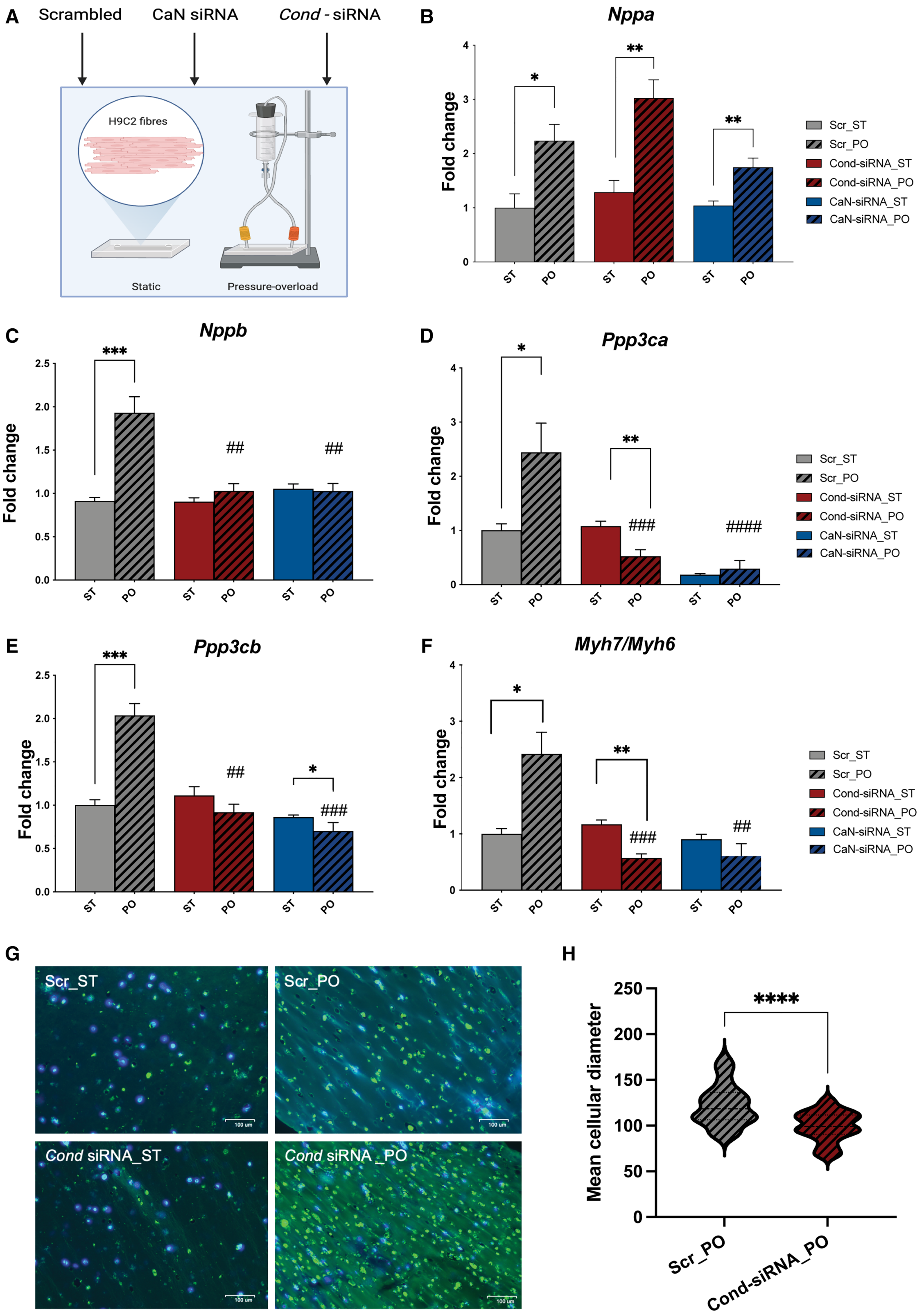

(legend on next page)

gene silencing observed only in CMs treated with the pro-hypertrophic stimulus PE, and not in other cell types. All *Cond*-siRNA constructs—including 1st G, 2nd G, and Chol versions—effectively reduced *Ppp3ca* expression in PE-treated NRVMs, confirming their conditional activation. The conditional silencing of CaN was associated with downstream inhibition of key signaling pathways, such as NFATc1 nuclear translocation and ERK1/2 phosphorylation. Importantly, the *Cond*-siRNA prevented the upregulation of hypertrophic markers such as *Nppa*, *Nppb*, *Mhy7/Myh6* ratio, and reduced CM area under phenylephrine-induced stress without affecting atrophy pathways at relevant concentrations. The observation that *Cond*-siRNA does not induce muscle atrophy markers, such as Murf1, within the therapeutic concentration range (1–10 nM) highlights its safety and specificity. This aligns with pharmacological studies showing that CaN inhibitors such as cyclosporin A suppress pathological cardiac hypertrophy without inducing skeletal muscle atrophy in short-term models.[46,47] These observations underscore the efficacy of *Cond*-siRNA in targeting central mediators of pathological hypertrophy while preserving physiological functions in unstressed cells.

The Chol *Cond*-siRNA PE-treatment was the most effective in reversing the increase in *Nppa* expression compared to other groups,[48] suggesting it may facilitate more rapid or efficient intracellular delivery and activation in response to hypertrophic signaling. On the other hand, the 2nd G *Cond*-siRNA appeared to have unexpected off-target effects on NFATc1. These observations highlight that specific chemistries for the *Cond*-siRNAs may ultimately be important in determining their therapeutic efficacy and off-target effects.

The preclinical HOC model further validated the stress-specific functionality of the 1st G *Cond*-siRNA. In this model, the activation of *Cond*-siRNA in response to PO effectively and selectively silenced the *Ppp3ca* CaN isoform, as evidenced by reductions in its expression levels and normalization of the *Myh7/Myh6* ratio. Unlike commercial CaN siRNAs, which showed silencing in both ST and PO conditions, *Cond*-siRNA demonstrated selective activity, reinforcing its potential for use in dynamic and tissue-specific disease contexts.

Several challenges remain to be addressed to advance such therapies or expand them into other indications. Most notably, ensuring tissue-specific activation, as demonstrated by our use of *Nppa* sensors for *Cond*-siRNA activation in CMs, for other cell types or disease states may require considerable work both in determining the appropriate disease-biomarker as well as the design of appropriate sensor strands for *Cond*-siRNA. While 1st G *Cond*-siRNA constructs demonstrated the most efficient conditional silencing of *Ppp3ca* (CaN), 2nd G and *Chol*-conjugated siRNA variants exhibited partial non-targeted effects, including reduced nuclear NFATc1 levels even in PBS-treated cells (in the absence of hypertrophic stimuli). This phenomenon, observed in our experiments, could not be attributed to CaN silencing or "leakiness" of the *Cond*-siRNA system, but may instead reflect off-target transcriptional modulation (e.g., of other CaN isoforms) or cellular stress responses triggered by specific siRNA constructs. Furthermore, while *Nppa* sensors proved effective in this study, their variability highlights the need for expanding RNA sensor repertoires or employing multiplexed strategies to enhance adaptability across diverse pathological scenarios. Robust *in vivo* validation, including testing in animal models to assess pharmacokinetics, safety, and efficacy under chronic conditions, remains essential. Advanced delivery systems, such as nanoparticles or CM-specific ligands, could improve tissue targeting and minimize degradation, while dosage optimization is crucial to prevent off-target effects such as upregulation of muscle atrophy markers. Future iterations of *Cond*-siRNA designs will require careful optimization of sensor-activator pairs and chemical modifications to mitigate non-specific interactions.

Our *Cond*-siRNA approach targets CaN specifically in CMs, but its potential systemic effects in HF should be considered. Even with CM-specific inhibition, CaN targeting could affect cardiovascular parameters, including hypertension due to altered renal sodium handling and vascular tone regulation due to low-level "release" of the guide RNA from the *Cond*-siRNA complex even in the absence of sensor binding.[49,50] Additionally, cardiac arrhythmias might arise from changes in calcium signaling, affecting ion channels and action potential duration.[22,51] Electrolyte imbalances, such as hyperkalemia, may result from impaired renal potassium excretion.[51–53] While systemic inhibitors such as cyclosporine and tacrolimus cause these effects broadly,[50,54] our targeted siRNA aims to reduce such risks. However, thorough preclinical evaluation of cardiovascular function and off-target effects will be crucial to assess any potential residual systemic effects. By addressing these challenges, *Cond*-siRNA constructs hold promise as a personalized therapeutic tool for HF, capable of targeting multiple pathological pathways with precision.

In summary, by enabling selective silencing under pathological conditions, *Cond*-siRNA approaches offer an unparalleled combination

Figure 5. Evaluation of *Cond*-siRNA in the HOC model under HF-like stress conditions
(A) Schematic representation of the heart-on-chip (HOC) model. H9c2 fibrin-based fibers were subjected to static (ST) or pressure-overload (PO) conditions (200/10 mmHg) following transfection with 20 nM scrambled siRNA (Scr), commercial calcineurin siRNA (CaN siRNA), or 1st generation *Cond*-siRNA (*Cond*-siRNA). (B–E) Expression levels of stress markers (B) *Nppa*, (C) *Nppb*, (D) *Ppp3ca*, and (E) *Ppp3cb* across experimental groups were estimated using RT-qPCR. (F) Expression levels of the *Myh7/Myh6* ratio, a marker of cardiac remodeling, across all groups assessed by RT-qPCR. All data are derived from experiments with $n = 3$ represented as mean (SD). Unpaired t test was performed between ST and PO groups, with significance indicated as $*p \leq 0.05$, $**p \leq 0.01$, and $***p \leq 0.001$; and between scrambled PO vs. other PO groups using ANOVA with significance indicated as $\#p \leq 0.05$, $\#\#p \leq 0.01$, and $\#\#\#p \leq 0.001$. (G) Wheat germ agglutinin (WGA) and DAPI staining of H9c2 fibers under static and pressure-overload conditions, represented with a scale bar of 100 μm. (H) Quantification of mean cellular diameters Scr_PO and *Cond*-siRNA_PO measured using ImageJ software. An unpaired t test was performed between the two groups, with significance indicated as $****p < 0.0001$.

of specificity and efficacy, minimizing off-target effects while effectively addressing hypertrophy. This methodology bridges critical gaps in current RNA-based therapies and represents a significant advancement in precision medicine for HF management. Future studies could expand the *Cond*-siRNA platform by integrating multiple constructs with distinct sensors and guide RNAs, targeting both hypertrophy and fibrosis to address the multifaceted nature of cardiac remodeling. This multiplexed approach would enable precise, condition-specific activation, improving therapeutic efficacy by tackling interconnected remodeling mechanisms, paving the way for personalized, multi-target therapies for HF while minimizing both off-target effects and undesired on-target effects in other cell types.

MATERIALS AND METHODS

Transverse aortic constriction model of non-ischemic HF

Anesthetized male mice aged 8–12 weeks underwent a lateral thoracotomy, followed by suture constriction of the transverse aorta against a blunted 25-gauge needle. Control sham operated mice underwent the same open chest surgery without constriction of the aorta. Heart tissues were harvested 6 weeks post-surgery for RNA isolation. All animal experiments were conducted under approval of the Institutional Animal Care and Use Committee.

Ischemia/reperfusion injury ischemic HF model

Ischemia/reperfusion (I/R) was generated by ligating the left anterior descending coronary artery (LAD) using a 7/0 silk thread for 20 min, followed by reperfusion, while control sham mice underwent the same process but without LAD ligation. Mice's hearts were harvested 6 weeks post-surgery for RNA isolation.

Annealing and purification of the *Cond*-siRNA

Equimolar concentrations of the sensor, guide and core strands were combined making a total volume of 20 μL in 1× PBS. The strands were annealed via thermal annealing (85°C for 3 min and 50°C for 1 h, followed by cool down). Annealed *Cond*-siRNA constructs were mixed with RNA Loading Dye (component of NEXTFLEX Small RNA-Seq Kit v3) and loaded in a 10% TBE gel (from Life Technologies, EC6275BOX). Gel was run in a XCell *SureLock* Mini-Cell (from Life Technologies, EI0001) in 1× TBE (Bio-Rad, 1610770), 120 V for 90 min. After staining the gel with SYBR Gold Nucleic Acid Gel Stain (10,000× concentrate in DMSO) (Thermo Fisher Scientific, S-11494), diluted 1× with 1× TBE, for 10 min, the band corresponding to the *Cond*-siRNA was cut over UV light. The band was pestle (USA Scientific, 1415-5390) crushed in 300 μL elution buffer (component of NEXTFLEX Small RNA-Seq Kit v3) and rotated for at least two hours at room temperature (RT). The eluted *Cond*-siRNA was filter in a Spin-X tube (Sigma-Aldrich, CLS8170-200EA), getting the eluate on the bottom, containing the *Cond*-siRNA.

Neonatal rat ventricular myocyte isolation

NRVMs were isolated from postnatal day 1 Wistar rat pups using collagenase II and pancreatin based enzymatic digestion, purified via Percoll gradient, and used for experiments 24 h post-isolation. NRVMs were cultured in DMEM (Life Technologies, cat. no. 11995073) supplemented with 10% horse serum (Thermo Fisher Scientific, cat. no. 26050-088), 5% fetal bovine serum (FBS) (Life Technologies, cat. no. 10437028), 1% penicillin-streptomycin (Thermo Fisher Scientific, cat. no. 15140122) and 1% L-glutamine (Thermo Fisher Scientific, cat. no. 25030-081).

Cond-siRNA and commercial siRNA transfection

Transfection of *Cond*-siRNA or other siRNA was performed using Lipofectamine RNAiMax Transfection Reagent (Thermo Fisher Scientific, cat. no. 13778150) into NRVMs in serum free DMEM media (Life Technologies, cat. no. 11995073), supplemented with 1% penicillin-streptomycin (Thermo Fisher Scientific, cat. no. 15140122) and 1% L-glutamine (Thermo Fisher Scientific, cat. no. 25030-081). As positive control of CaN silencing, a commercial ppp3ca siRNA was utilized (Thermo Fisher Scientific, cat. no. 162268). As negative control, AllStars Negative Control siRNA was used (QIAGEN, cat. no. 1022076). As positive control of muscle atrophy, miR-29b mimic was used (Thermo Fisher Scientific, cat. no. 4464066) at a 50 pmol concentration.

RNA isolation and RT-qPCR

For cellular RNA isolation, NRVMs, NRCFs, and Jurkat T cells were initially lysed with Trizol, and consequently RNA was isolated via chloroform-isopropanol-ethanol protocol. cDNA was then prepared using the High Capacity cDNA Reverse Transcription Kit (Thermo Fisher Scientific, cat. no. 43-688-13), followed by RT-qPCR through the Kapa Sybr Green system for qPCR (Thermo Fisher Scientific, cat. no. KK2601). The sequences of the primers used for quantifying each mRNA are indicated as follows: *Nppa* forward 5′- GTGC GGTGTCCAACACAGAT-3′, reverse 5′- TCCAATCCTGTCAATC CTACCC-3′; *Nppb* forward 5′-ACAGCTCTCAAAGGACCAAG -3′, reverse 5′- GCTTGAACTATGTGCCATCTTG -3′; *Myh7* forward 5′-CCATCTCTGACAACGCCTATC -3′, reverse 5′- TCTT GGTGTTGACGGTCTTAC -3′; *Myh6* forward 5′- CCATCCTCA TCACTGGAGAATC -3′, reverse 5′- GGTGCCCTTGTTTGCAT TAG -3′; *Mef2c* forward 5′- TCTCCGCGTTCTTATCCCAC -3′, reverse 5′- AGGAGTTGCTACGGAAACCAC -3′; *Myocd* forward 5′- GATGGGCTCTCTCCAGATCAG-3′, reverse 5′-GGCTGCATC ATTCTTGTCACTT -3′; *Ddit4* forward 5′- CCAGTTCGCTCAC CCTTC -3′, reverse 5′- GAAACGATCCCAGAGGCTAG-3′; *Ppp3ca* forward 5′-TTCAGAACGCGTTTATGACGCCT-3′, reverse 5′-CC TGATGACCTCCTTCCGGG -3′; *Ppp3cb* forward 5′- CCATACTT AGGCGGGAGAAAAC -3′, reverse 5′- AAGGTATCGTGTATTAG CAGGTG -3′; *Murf1* forward 5′- GAACGACCGAGTTCAGACT ATC -3′, reverse 5′- CCTCCTCCTCCTCTTCAGTAA -3′; *Ppp3cb* (for siRNA duplexes 6516 and 6517) forward 5′- GCTCAAG ATGCAGGCTATAGAA -3′, reverse 5′- CCAACAAATGGTAAA GACCATGTAA -3′; *Ppp3cb* (for siRNA duplex 6518) forward 5′- TGTTGCCTAGTGGAGTGTTG -3′, reverse 5′- CCGTGGTTCTC AGTGGTATG -3′; *Ppp3cb* (for siRNA duplex 6519) forward 5′- TTAATGTGGAACCTCCCTCACC -3′, reverse 5′- TTAATGTG GAACCTCCCTCACC -3′; *Ppp3cb* (for siRNA duplexes 6520 and

6521) forward 5′- GAGGATGGATTAGCATGGTC -3′, reverse 5′- TGGATCTTGTTCAAGTAAGAGT -3′; *Actb* forward 5′- GTGAC GTTGACATCCGTAAAGA - 3′, reverse 5′ - GCCGGACTCATCG TACTCC - 3′.

Ppp3ca (*human*) forward 5′- TGGATGTTCTTGCCTCTGAC -3′, reverse 5′- TGTTTAATCACCATCCCCACC -3′; *Nppa* (*human*) forward 5′- AACGCAGACCTGATGGATTT -3′, reverse 5′- TCC TCCCTGGCTGTTATCT -3′; *Hprt1* (*human*) forward 5′- TTGCTG ACCTGCTGGATTAC -3′, reverse 5′- CTTGCGACCTTGACCA TCTT -3′. mRNA quantification was normalized to the housekeeping gene β-actin (mouse and rat) or HPRT (human) and represented as fold change ($2^{-\Delta\Delta^{Ct}}$) versus the respective control condition depending on the experimental set up.

Protein isolation and western blotting

Cytoplasmic and nuclear proteins from NRVMs were extracted through the Thermo Scientific NE PER Nuclear and Cytoplasmic Extraction Kit (Thermo Fisher Scientific, cat. no. 78833). The Pierce BCA protein assay (Thermo Fisher Scientific, cat. no. 23227) was performed to quantify lysates' protein concentration, and 20 μg of each sample were used for 4%–20% SDS-PAGE electrophoresis. Gels were transferred to polyvinylidene fluoride or membranes (Bio-Rad) and blocked with 5% BSA for 1 h at RT. Primary antibodies were incubated ON at 4°C rocking at a 1:1,000 concentration. The primary antibodies used were the following ones: CaN (Cell Signaling Technology, cat. no. 2614S), NFATc1 NFATc1(Sigma-Aldrich, cat. no. SAB2101576), MYH7 (Santa Cruz Biotechnology, cat. no. sc-53089), phospho p44/42 MAPK (Erk1/2) (Thr202/Tyr204) (Cell Signaling Technology, cat. no. 4370S), and P44/42 MAPK (Erk1/2) (137F5) (Cell Signaling Technology, cat. no. 4695S). Secondary HRP-antibodies (Agilent) were incubated for 1 h at RT rocking. Blots were developed using the Supersignal Femto developer (Thermo Fisher Scientific, cat. no. 34095).

Immunofluorescence

NRVMs treated with the *Cond*-siRNA were formalin-fixed for 10 min, permeabilized with 0.5% Triton X-100 in PBS, and blocked for 1 h at RT. Staining with TroponinT antibody (Thermo Fisher Scientific, cat. no. MA5-12960) (1:250) was performed overnight at 4°C, followed by 2 h incubation at RT with the secondary antibody (1:250), and mounted with DAPI containing mounting media (Invitrogen, cat. no. P36935). Images were taken with a Leica SP8 confocal microscope, and cell area quantification was performed through ImageJ.

Live imaging

NRVMs, NRCFs, and Jurkat cells were transfected with *Cond*-siRNA conjugated with Alexa Fluor 594 with the appropriate transfection agents (Lipofectamine RNAimax for NRVM and NRCFs; Attractene [QIAGEN] for the Jurkat) and after 48 h, stained with DAPI (Invitrogen, cat. no. D1306) for 10 min. The cells were then imaged using the Bio-Rad ZOE Fluorescent Cell Imager and quantified using ImageJ.

Heart-on-chip model

HOC devices were fabricated via soft lithography using PDMS mixed with 10:1 base-to-crosslinker ratio, featuring a bottom flexible membrane, 3 pairs of anchored posts, attached to a ~1 cm-tall frame via oxygen plasma bonding, to create a chamber that holds 3 engineered tissues and 4 mL media. H9c2 cells were cultured until 60%–70% confluent, at which point the media in each well of a 6-well plate is replaced with 2 mL OptiMEM. After 2 h, cells were transfected with 20 nM of siRNA following the manufacturer's protocol (Lipofectamine). Eight hours post-transfection, the media is switched to DMEM containing 1% P/S (no FBS). Twenty-four hours later, cells are dissociated to create fibers using a mixture of 33 mg/mL fibrinogen (84 μL) and 25 U/mL thrombin (16 μL) per fiber. Once gelled, the fibers are rested in DMEM with 1% P/S and 10% FBS for 48 h. Following this static culture, the media is changed to serum-free DMEM with 1% P/S for 24 h prior to experimentation. During the experiment, fibers in the PO group are placed in a 200/10 mmHg loop for 24 h in serum-free media, while fibers in the static group remain in serum-free media under ST.

Statistical analysis

GraphPad Prism software was used to perform the statistical tests. Comparisons between the two groups were conducted using an independent two-sample t test. Differences among three or more groups were analyzed using one-way analysis of variance (ANOVA) with Tukey's post hoc test for pairwise comparisons.

DATA AND CODE AVAILABILITY

The data generated in this study are availbale on request from the corresponding author.

ACKNOWLEDGMENTS

A.M.S. and P.G. were supported by AHA postdoctoral fellowships 18POST34030167 (A.M.S.) and 23POST1014230 (P.G.). C.G. was supported by T32 EB023872. S.D. was supported by NHLBI 1R35HL150807. HOC. experiments were funded by NIH R01 grant no. HL148462.

AUTHOR CONTRIBUTIONS

P.G. and A.M.S. designed and performed experiments, analyzed and interpreted the data, wrote the manuscript, and prepared the figures. C.G., G.L., S.-p.H., L.S., and R.K. performed experiments. C.A. and M.S. edited the manuscript. P.S., J.R., W.A.G., and S.D. supplied funding, were involved in the study design, and supervised the work. All authors read and approved the final manuscript.

DECLARATION OF INTERESTS

S.D., J.R., W.A.G., S.-p.H., and L.S. are co-founders of Switch Therapeutics and hold equity in the company. Switch was not involved in the design, conduct, or funding of any part of the study but does license IP on conditional siRNAs.

SUPPLEMENTAL INFORMATION

Supplemental information can be found online at https://doi.org/10.1016/j.omtn.2025.102667.

REFERENCES

1. Benjamin, E.J., Virani, S.S., Callaway, C.W., Chamberlain, A.M., Chang, A.R., Cheng, S., Chiuve, S.E., Cushman, M., Delling, F.N., Deo, R., et al. (2018). Heart Disease and Stroke Statistics-2018 Update: A Report From the American Heart Association. Circulation *137*, e67–e492. https://doi.org/10.1161/CIR.0000000000000558.

2. Khan, M.S., Shahid, I., Bennis, A., Rakisheva, A., Metra, M., and Butler, J. (2024). Global epidemiology of heart failure. Nat. Rev. Cardiol. *21*, 717–734. https://doi.org/10.1038/s41569-024-01046-6.

3. 2024 Heart Disease and Stroke Statistics: A Report of US and Global Data From the American Heart Association. https://www.ahajournals.org/doi/epub/10.1161/CIR.0000000000001209.

4. Joglar, J.A., Chung, M.K., Armbruster, A.L., Benjamin, E.J., Chyou, J.Y., Cronin, E. M., Deswal, A., Eckhardt, L.L., Goldberger, Z.D., Gopinathannair, R., et al. (2024). 2023 ACC/AHA/ACCP/HRS Guideline for the Diagnosis and Management of Atrial Fibrillation: A Report of the American College of Cardiology/American Heart Association Joint Committee on Clinical Practice Guidelines. Circulation *149*, e1–e156. https://doi.org/10.1161/CIR.0000000000001193.

5. McDonagh, T.A., Metra, M., Adamo, M., Gardner, R.S., Baumbach, A., Böhm, M., Burri, H., Butler, J., Čelutkienė, J., Chioncel, O., et al. (2023). 2023 Focused Update of the 2021 ESC Guidelines for the diagnosis and treatment of acute and chronic heart failure: Developed by the task force for the diagnosis and treatment of acute and chronic heart failure of the European Society of Cardiology (ESC) With the special contribution of the Heart Failure Association (HFA) of the ESC. Eur. Heart J. *44*, 3627–3639. https://doi.org/10.1093/eurheartj/ehad195.

6. Kehat, I., and Molkentin, J.D. (2010). Molecular Pathways Underlying Cardiac Remodeling During Pathophysiological Stimulation. Circulation *122*, 2727–2735. https://doi.org/10.1161/CIRCULATIONAHA.110.942268.

7. Frey, N., and Olson, E.N. (2003). Cardiac hypertrophy: the good, the bad, and the ugly. Annu. Rev. Physiol. *65*, 45–79. https://doi.org/10.1146/annurev.physiol.65.092101.142243.

8. Paulus, W.J., and Tschöpe, C. (2013). A Novel Paradigm for Heart Failure With Preserved Ejection Fraction: Comorbidities Drive Myocardial Dysfunction and Remodeling Through Coronary Microvascular Endothelial Inflammation. J. Am. Coll. Cardiol. *62*, 263–271. https://doi.org/10.1016/j.jacc.2013.02.092.

9. Pfeffer, M.A., and Braunwald, E. (1990). Ventricular remodeling after myocardial infarction. Experimental observations and clinical implications. Circulation *81*, 1161–1172. https://doi.org/10.1161/01.cir.81.4.1161.

10. Zile, M.R. (2003). Heart failure with preserved ejection fraction: is this diastolic heart failure? J. Am. Coll. Cardiol. *41*, 1519–1522. https://doi.org/10.1016/s0735-1097(03)00186-4.

11. Drazner, M.H., Dries, D.L., Peshock, R.M., Cooper, R.S., Klassen, C., Kazi, F., Willett, D., and Victor, R.G. (2005). Left ventricular hypertrophy is more prevalent in blacks than whites in the general population: the Dallas Heart Study. Hypertension *46*, 124–129. https://doi.org/10.1161/01.HYP.0000169972.96201.8e.

12. Maron, B.J., Ommen, S.R., Semsarian, C., Spirito, P., Olivotto, I., and Maron, M.S. (2014). Hypertrophic cardiomyopathy: present and future, with translation into contemporary cardiovascular medicine. J. Am. Coll. Cardiol. *64*, 83–99. https://doi.org/10.1016/j.jacc.2014.05.003.

13. Sikand, N., Stendahl, J., Sen, S., Lampert, R., and Day, S. (2025). Current management of hypertrophic cardiomyopathy. BMJ *389*, e077274. https://doi.org/10.1136/bmj-2023-077274.

14. Santos Mateo, J.J., Sabater Molina, M., and Gimeno Blanes, J.R. (2018). Hypertrophic cardiomyopathy. Med. Clín. *150*, 434–442. https://doi.org/10.1016/j.medcle.2017.09.029.

15. Maurizi, N., Michels, M., Rowin, E.J., Semsarian, C., Girolami, F., Tomberli, B., Cecchi, F., Maron, M.S., Olivotto, I., and Maron, B.J. (2019). Clinical Course and Significance of Hypertrophic Cardiomyopathy Without Left Ventricular Hypertrophy. Circulation *139*, 830–833. https://doi.org/10.1161/CIRCULATIONAHA.118.037264.

16. Burchfield, J.S., Xie, M., and Hill, J.A. (2013). Pathological ventricular remodeling: mechanisms: part 1 of 2. Circulation *128*, 388–400. https://doi.org/10.1161/CIRCULATIONAHA.113.001878.

17. Mann, D.L., and Bristow, M.R. (2005). Mechanisms and models in heart failure: the biomechanical model and beyond. Circulation *111*, 2837–2849. https://doi.org/10.1161/CIRCULATIONAHA.104.500546.

18. van Berlo, J.H., Maillet, M., and Molkentin, J.D. (2013). Signaling effectors underlying pathologic growth and remodeling of the heart. J. Clin. Investig. *123*, 37–45. https://doi.org/10.1172/JCI62839.

19. Da Costa Martins, P.A., and De Windt, L.J. (2012). MicroRNAs in control of cardiac hypertrophy. Cardiovasc. Res. *93*, 563–572. https://doi.org/10.1093/cvr/cvs013.

20. Nakamura, M., and Sadoshima, J. (2018). Mechanisms of physiological and pathological cardiac hypertrophy. Nat. Rev. Cardiol. *15*, 387–407. https://doi.org/10.1038/s41569-018-0007-y.

21. Frey, N., Katus, H.A., Olson, E.N., and Hill, J.A. (2004). Hypertrophy of the Heart. Circulation *109*, 1580–1589. https://doi.org/10.1161/01.CIR.0000120390.68287.BB.

22. Molkentin, J.D., Lu, J.R., Antos, C.L., Markham, B., Richardson, J., Robbins, J., Grant, S.R., and Olson, E.N. (1998). A calcineurin-dependent transcriptional pathway for cardiac hypertrophy. Cell *93*, 215–228. https://doi.org/10.1016/S0092-8674(00)81573-1.

23. Olson, E.N., and Williams, R.S. (2000). Calcineurin signaling and muscle remodeling. Cell *101*, 689–692. https://doi.org/10.1016/s0092-8674(00)80880-6.

24. Berry, J.M., Le, V., Rotter, D., Battiprolu, P.K., Grinsfelder, B., Tannous, P., Burchfield, J.S., Czubryt, M., Backs, J., Olson, E.N., et al. (2011). Reversibility of adverse, calcineurin-dependent cardiac remodeling. Circ. Res. *109*, 407–417. https://doi.org/10.1161/CIRCRESAHA.110.228452.

25. Dutta, D., Barr, V.A., Akpan, I., Mittelstadt, P.R., Singha, L.I., Samelson, L.E., and Ashwell, J.D. (2017). Recruitment of calcineurin to the TCR positively regulates T cell activation. Nat. Immunol. *18*, 196–204. https://doi.org/10.1038/ni.3640.

26. Han, S., Scherer, L., Gethers, M., Salvador, A.M., Salah, M.B.H., Mancusi, R., Sagar, S., Hu, R., DeRogatis, J., Kuo, Y.-H., et al. (2022). Programmable siRNA pro-drugs that activate RNAi activity in response to specific cellular RNA biomarkers. Mol. Ther. Nucleic Acids *27*, 797–809. https://doi.org/10.1016/j.omtn.2021.12.039.

27. Zadeh, J.N., Steenberg, C.D., Bois, J.S., Wolfe, B.R., Pierce, M.B., Khan, A.R., Dirks, R.M., and Pierce, N.A. (2011). NUPACK: Analysis and design of nucleic acid systems. J. Comput. Chem. *32*, 170–173. https://doi.org/10.1002/jcc.21596.

28. Molkentin, J.D. (2000). Calcineurin and Beyond. Circ. Res. *87*, 731–738. https://doi.org/10.1161/01.RES.87.9.731.

29. Taigen, T., De Windt, L.J., Lim, H.W., and Molkentin, J.D. (2000). Targeted inhibition of calcineurin prevents agonist-induced cardiomyocyte hypertrophy. Proc *97*, 1196–1201. https://doi.org/10.1073/pnas.97.3.1196.

30. Luo, Y., Jiang, N., May, H.I., Luo, X., Ferdous, A., Schiattarella, G.G., Chen, G., Li, Q., Li, C., Rothermel, B.A., et al. (2021). Cooperative Binding of ETS2 and NFAT Links Erk1/2 and Calcineurin Signaling in the Pathogenesis of Cardiac Hypertrophy. Circulation *144*, 34–51. https://doi.org/10.1161/CIRCULATIONAHA.120.052384.

31. Molkentin, J.D. (2004). Calcineurin–NFAT signaling regulates the cardiac hypertrophic response in coordination with the MAPKs. Cardiovasc. Res. *63*, 467–475. https://doi.org/10.1016/j.cardiores.2004.01.021.

32. Li, J., Chan, M.C., Yu, Y., Bei, Y., Chen, P., Zhou, Q., Cheng, L., Chen, L., Ziegler, O., Rowe, G.C., et al. (2017). miR-29b contributes to multiple types of muscle atrophy. Nat. Commun. *8*, 15201. https://doi.org/10.1038/ncomms15201.

33. Donoghue, L., Graham, C., and Sethu, P. (2023). Acute Response of Engineered Cardiac Tissue to Pressure and Stretch. Cells Tissues Organs *212*, 352–362. https://doi.org/10.1159/000525250.

34. Bazgir, F., Nau, J., Nakhaei-Rad, S., Amin, E., Wolf, M.J., Saucerman, J.J., Lorenz, K., and Ahmadian, M.R. (2023). The Microenvironment of the Pathogenesis of Cardiac Hypertrophy. Cells *12*, 1780. https://doi.org/10.3390/cells12131780.

35. Lu, D., and Thum, T. (2019). RNA-based diagnostic and therapeutic strategies for cardiovascular disease. Nat. Rev. Cardiol. *16*, 661–674. https://doi.org/10.1038/s41569-019-0218-x.

36. Huang, C.-K., Kafert-Kasting, S., and Thum, T. (2020). Preclinical and Clinical Development of Noncoding RNA Therapeutics for Cardiovascular Disease. Circ. Res. *126*, 663–678. https://doi.org/10.1161/CIRCRESAHA.119.315856.

37. Kowara, M., Borodzicz-Jazdzyk, S., Rybak, K., Kubik, M., and Cudnoch-Jedrzejewska, A. (2021). Therapies Targeted at Non-Coding RNAs in Prevention and Limitation of Myocardial Infarction and Subsequent Cardiac Remodeling—Current Experience and Perspectives. Int. J. Mol. Sci. *22*, 5718. https://doi.org/10.3390/ijms22115718.

38. Zaleta-Rivera, K., Dainis, A., Ribeiro, A.J.S., Cordero, P., Rubio, G., Shang, C., Liu, J., Finsterbach, T., Parikh, V.N., Sutton, S., et al. (2019). Allele-Specific Silencing Ameliorates Restrictive Cardiomyopathy Attributable to a Human Myosin

Regulatory Light Chain Mutation. Circulation *140*, 765–778. https://doi.org/10.1161/CIRCULATIONAHA.118.036965.

39. Kumarswamy, R., and Thum, T. (2013). Non-coding RNAs in Cardiac Remodeling and Heart Failure. Circ. Res. *113*, 676–689. https://doi.org/10.1161/CIRCRESAHA.113.300226.

40. Li, J., Salvador, A.M., Li, G., Valkov, N., Ziegler, O., Yeri, A., Yang Xiao, C., Meechoovet, B., Alsop, E., Rodosthenous, R.S., et al. (2021). Mir-30d Regulates Cardiac Remodeling by Intracellular and Paracrine Signaling. Circ. Res. *128*, e1–e23. https://doi.org/10.1161/CIRCRESAHA.120.317244.

41. Bao, J., Lu, Y., She, Q., Dou, W., Tang, R., Xu, X., Zhang, M., Zhu, L., Zhou, Q., Li, H., et al. (2021). MicroRNA-30 regulates left ventricular hypertrophy in chronic kidney disease. JCI Insight *6*, e138027. https://doi.org/10.1172/jci.insight.138027.

42. Callis, T.E., Pandya, K., Seok, H.Y., Tang, R.-H., Tatsuguchi, M., Huang, Z.-P., Chen, J.-F., Deng, Z., Gunn, B., Shumate, J., et al. (2009). MicroRNA-208a is a regulator of cardiac hypertrophy and conduction in mice. J. Clin. Investig. *119*, 2772–2786. https://doi.org/10.1172/JCI36154.

43. Sussman, M.A., Lim, H.W., Gude, N., Taigen, T., Olson, E.N., Robbins, J., Colbert, M.C., Gualberto, A., Wieczorek, D.F., and Molkentin, J.D. (1998). Prevention of cardiac hypertrophy in mice by calcineurin inhibition. Science *281*, 1690–1693. https://doi.org/10.1126/science.281.5383.1690.

44. Olson, E.N., and Molkentin, J.D. (1999). Prevention of Cardiac Hypertrophy by Calcineurin Inhibition. Circ. Res. *84*, 623–632. https://doi.org/10.1161/01.RES.84.6.623.

45. Anthony, C., Imran, M., Pouliopoulos, J., Emmanuel, S., Iliff, J.W., Moffat, K.J., Ross, J., Graham, R.M., Kotlyar, E., Muthiah, K., et al. (2021). Everolimus for the Prevention of Calcineurin-Inhibitor-Induced Left Ventricular Hypertrophy After Heart Transplantation (RADTAC Study). JACC: Heart Failure *9*, 301–313. https://doi.org/10.1016/j.jchf.2021.01.007.

46. Rothermel, B.A., McKinsey, T.A., Vega, R.B., Nicol, R.L., Mammen, P., Yang, J., Antos, C.L., Shelton, J.M., Bassel-Duby, R., Olson, E.N., et al. (2001). Myocyte-enriched calcineurin-interacting protein, MCIP1, inhibits cardiac hypertrophy in vivo. Proc *98*, 3328–3333. https://doi.org/10.1073/pnas.041614798.

47. Aoki, M.S., Miyabara, E.H., Soares, A.G., Salvini, T.F., and Moriscot, A.S. (2006). Cyclosporin-A does not affect skeletal muscle mass during disuse and recovery. Braz. J. Med. Biol. Res. *39*, 243–251. https://doi.org/10.1590/S0100-879X2006000200011.

48. Tokudome, T., Horio, T., Kishimoto, I., Soeki, T., Mori, K., Kawano, Y., Kohno, M., Garbers, D.L., Nakao, K., and Kangawa, K. (2005). Calcineurin–Nuclear Factor of Activated T Cells Pathway–Dependent Cardiac Remodeling in Mice Deficient in Guanylyl Cyclase A, a Receptor for Atrial and Brain Natriuretic Peptides. Circulation *111*, 3095–3104. https://doi.org/10.1161/CIRCULATIONAHA.104.510594.

49. Blankenstein, K.I., Borschewski, A., Labes, R., Paliege, A., Boldt, C., McCormick, J.A., Ellison, D.H., Bader, M., Bachmann, S., and Mutig, K. (2017). Calcineurin inhibitor cyclosporine A activates renal Na-K-Cl cotransporters via local and systemic mechanisms. Am. J. Physiol. Ren. Physiol. *312*, F489–F501. https://doi.org/10.1152/ajprenal.00575.2016.

50. Hoorn, E.J., Walsh, S.B., McCormick, J.A., Fürstenberg, A., Yang, C.-L., Roeschel, T., Paliege, A., Howie, A.J., Conley, J., Bachmann, S., et al. (2011). The calcineurin inhibitor tacrolimus activates the renal sodium chloride cotransporter to cause hypertension. Nat. Med. *17*, 1304–1309. https://doi.org/10.1038/nm.2497.

51. Perrier, E., Perrier, R., Richard, S., and Bénitah, J.-P. (2004). Ca2+ controls functional expression of the cardiac K+ transient outward current via the calcineurin pathway. J. Biol. Chem. *279*, 40634–40639. https://doi.org/10.1074/jbc.M407470200.

52. Duan, X.-P., Zhang, C.-B., Wang, W.-H., and Lin, D.-H. (2024). Role of calcineurin in regulating renal potassium (K+) excretion: Mechanisms of calcineurin inhibitor-induced hyperkalemia. Acta Physiol. *240*, e14189. https://doi.org/10.1111/apha.14189.

53. Xu, N., Hirohama, D., Ishizawa, K., Chang, W.X., Shimosawa, T., Fujita, T., Uchida, S., and Shibata, S. (2017). Hypokalemia and Pendrin Induction by Aldosterone. Hypertension *69*, 855–862. https://doi.org/10.1161/HYPERTENSIONAHA.116.08519.

54. Mohebbi, N., Mihailova, M., and Wagner, C.A. (2009). The calcineurin inhibitor FK506 (tacrolimus) is associated with transient metabolic acidosis and altered expression of renal acid-base transport proteins. Am. J. Physiol. Renal Physiol. *297*, F499–F509. https://doi.org/10.1152/ajprenal.90489.2008.

OMTN, Volume *36*

Supplemental information

Development of conditional-siRNA programmable riboswitch for targeting adverse cardiac remodeling

Priyanka Gokulnath, Ane M. Salvador, Caleb Graham, Si-ping Han, Guoping Li, Ramaswamy Kannappan, Christopher Azzam, Michail Spanos, Lisa Scherer, Palaniappan Sethu, John Rossi, William A. Goddard III, and Saumya Das

Supplemental Material

Supplemental Figures – Figure S1-S7

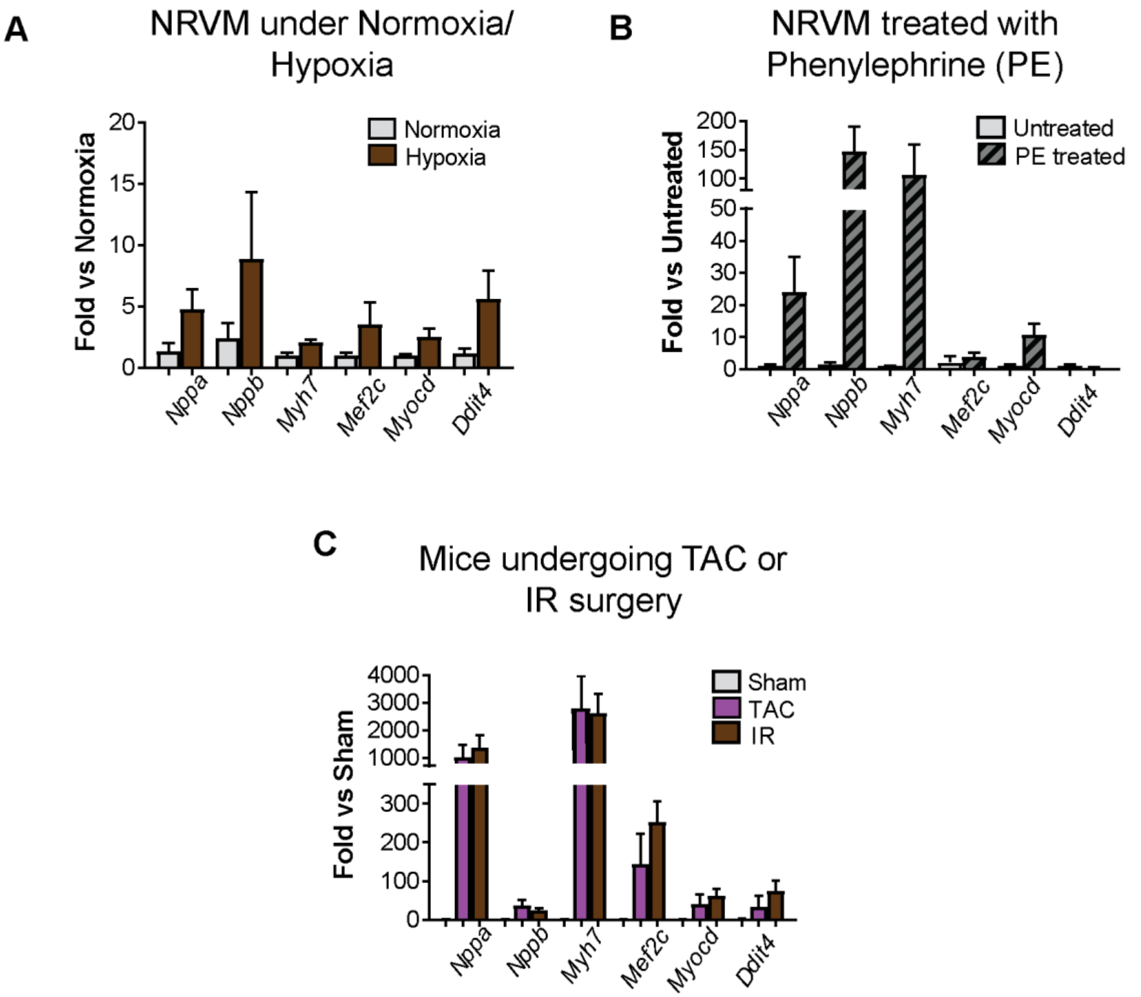

Figure S1. Identification of sensor RNA biomarkers upregulated in response to cardiac stress. Quantification of mRNA levels of cardiomyocyte pathology markers in **A-B**. NRVMs under stress or in mouse hearts **C**. undergoing TAC or IR surgeries. NRVM= Neonatal Rat Ventricular Myocytes, TAC= Transverse Aortic Constriction, IR= Ischemia Reperfusion injury, *Nppa*= Atrial Natriuretic Peptide, *Nppb*= Brain Natriuretic Peptide, *Myh7*= Myosin Heavy Chain beta isoform, *Mef2c*= Myocyte Enhancer factor 2C, *Myocd*= myocardin, *Ddit4*= DNA-damage-inducible transcript 4. Data are shown as Mean (SD) and derived from experiments with n=3.

A **Sensor:**

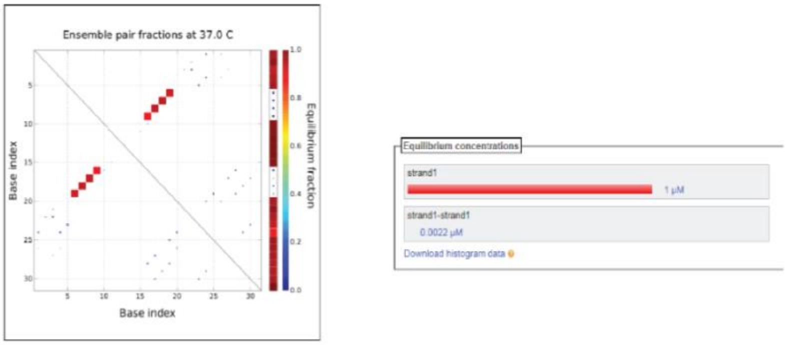

B **Sensor + Core:**

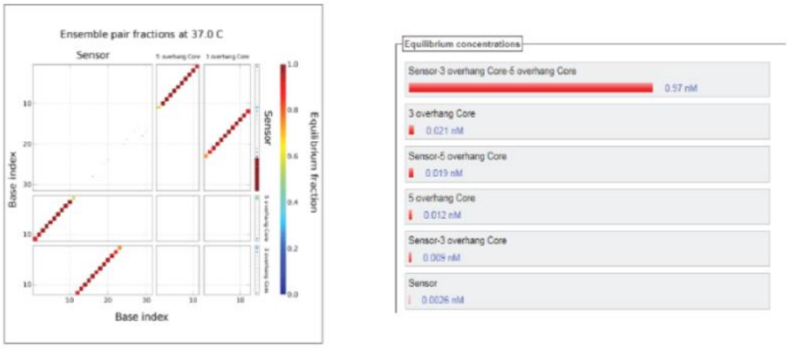

C **Guide + Core:**

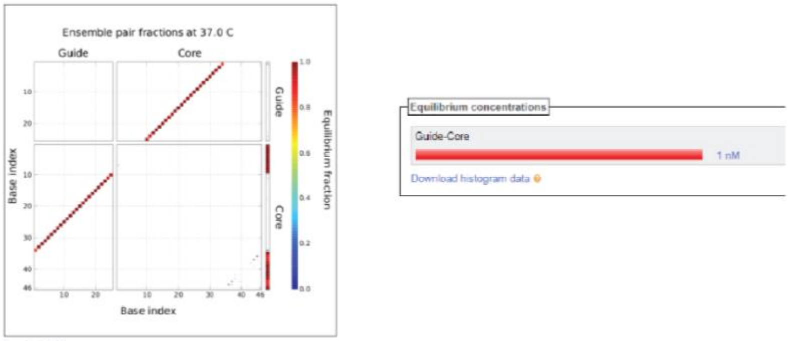

Figure S2. A-C. Nupack software's plots ranking the thermodynamic stability of the complexes formed between the different strands of the *Cond*-siRNA.

A

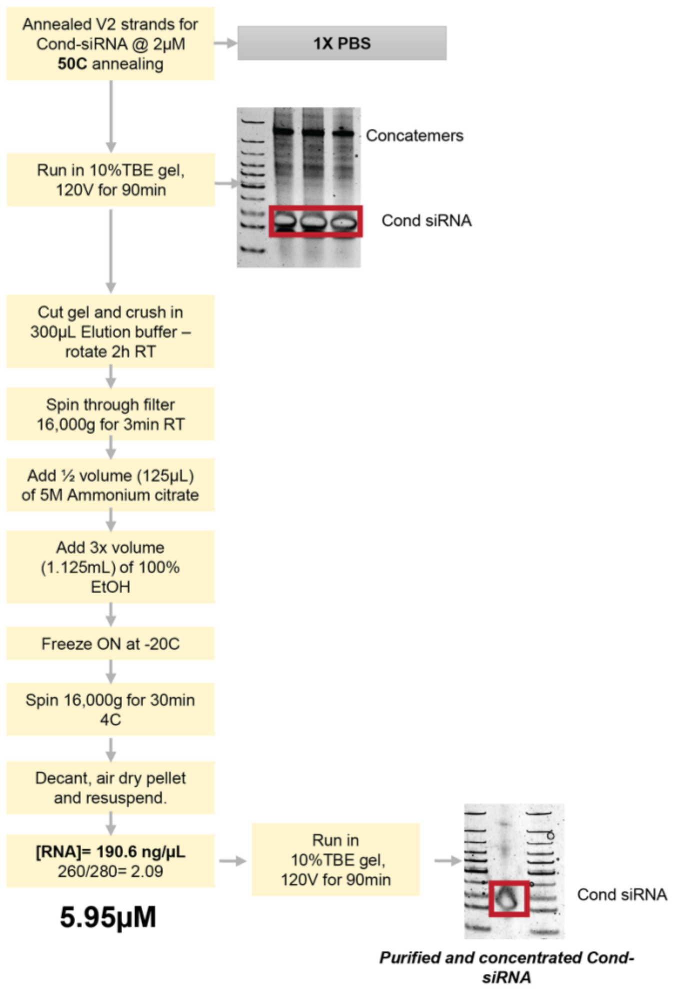

B

Re-annealing :

1. Bring purified construct to 1X PBS adding 10X PBS
2. 25°C to 50°C at 0.1C/s
3. 50°C for 30min
4. Back to 25°C at 0.1C/s

Figure S3. A. Protocol for strand annealing at high concentration, followed by purification to obtain the well-assembled Cond-siRNA and subsequent concentration. **B.** Protocol for re-annealing.

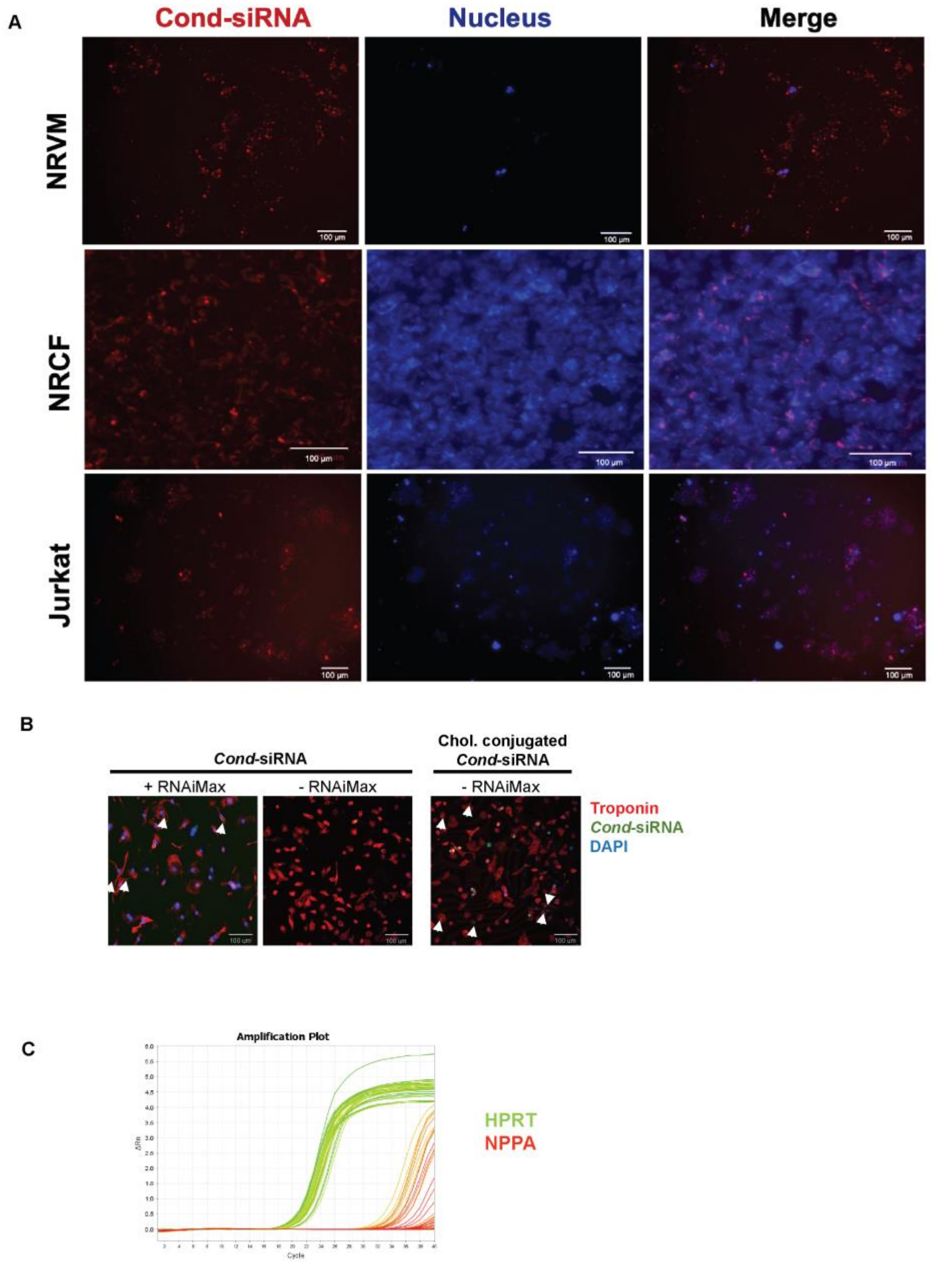

Figure S4. **A**. Representative images showing successful transfection of *Cond*-siRNA (red) in NRVM, NRCFs, and Jurkat cells with DAPi nuclear staining (blue) and Merged images at scale 100 µm. **B**. Representative images of liposomal RNAimax-mediated transfection and direct cholesterol-conjugated Cond-siRNA transfection of NRVMs. *Cond*-siRNA is labeled with FITC, and NRVMs are stained with Troponin T antibody (red) and DAPi nuclear staining (blue). **C.** Representative amplification plot of *Nppa* and *Hprt* (house-keeping gene) in Jurkat cell qRT-PCR.

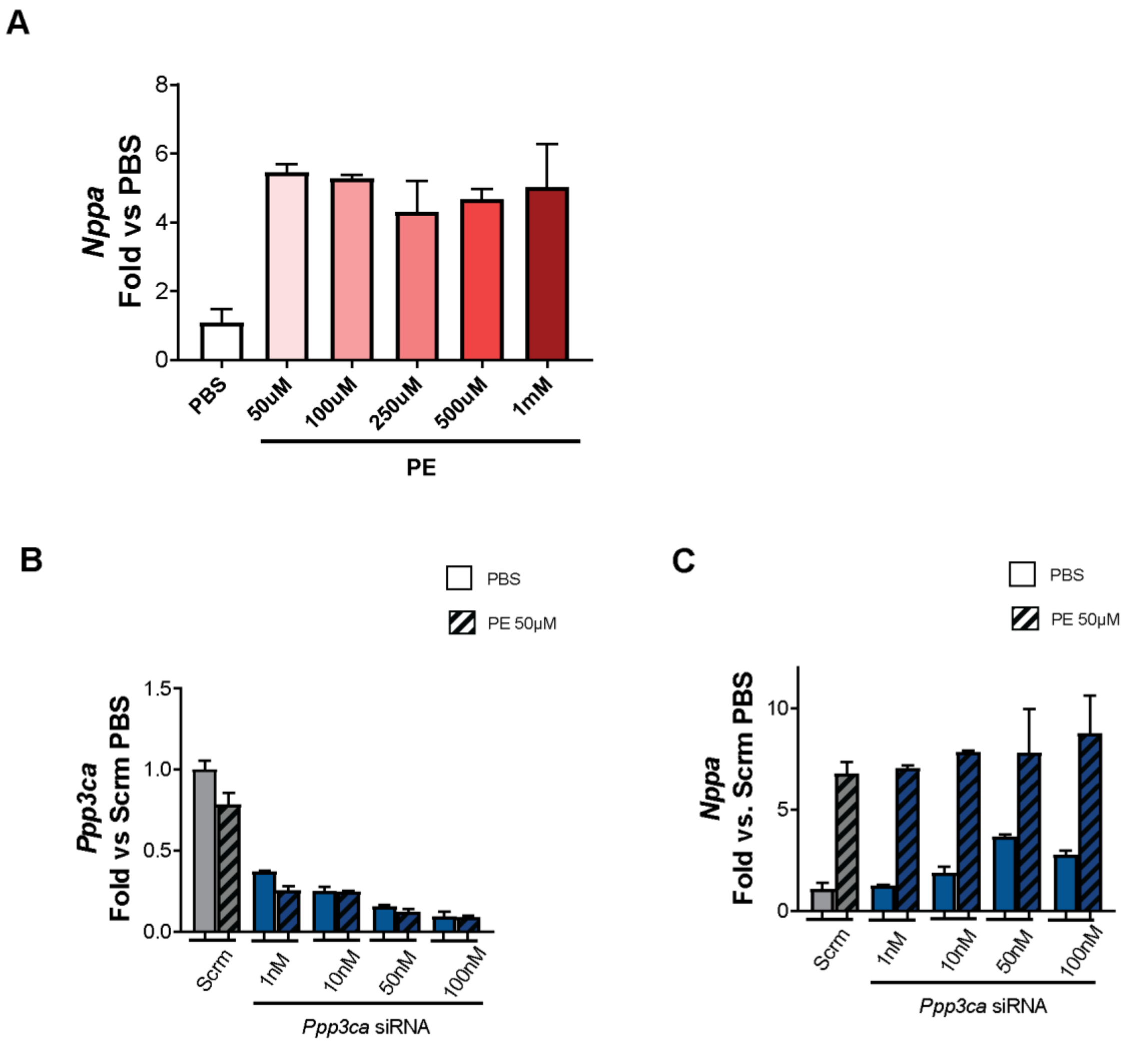

Figure S5. **A.** *Nppa* expression levels in NRVM treated with increasing concentrations of PE for 48 hrs. Data is represented as Fold change normalized to PBS (Fold vs PBS) as mean (SD). **B.** Calcineurin *Ppp3ca* isoform and **C.** *Nppa* mRNA expression in NRVMs transfected with increasing concentrations of commercial siRNA targeting *Ppp3ca*, and treated with 50μM PE. Data is represented as fold change normalized to Scrambled PBS (Fold vs. Scrm PBS) and derived from experiments with n=3 expressed as mean (SD).

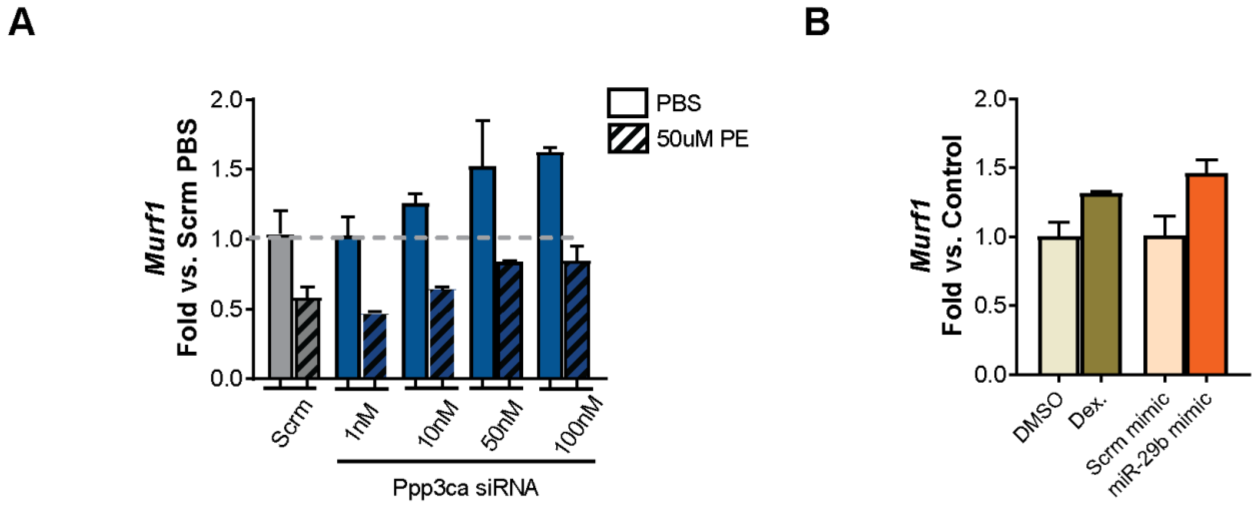

Figure S6. **A.** *Murf1* expression levels in NRVM treated with increasing concentrations of commercial siRNA and PE. Data is represented as fold change normalized to PBS (Fold vs PBS), expressed as mean (SD). **B.** *Murf1* expression levels in NRVM treated with 50μM dexamethasone for 24h or transfected with 50 pmol miR-29b mimic for 48h. Data is represented as fold change normalized to its respective control (Fold vs. control) and derived from experiments with n=3 as mean (SD).

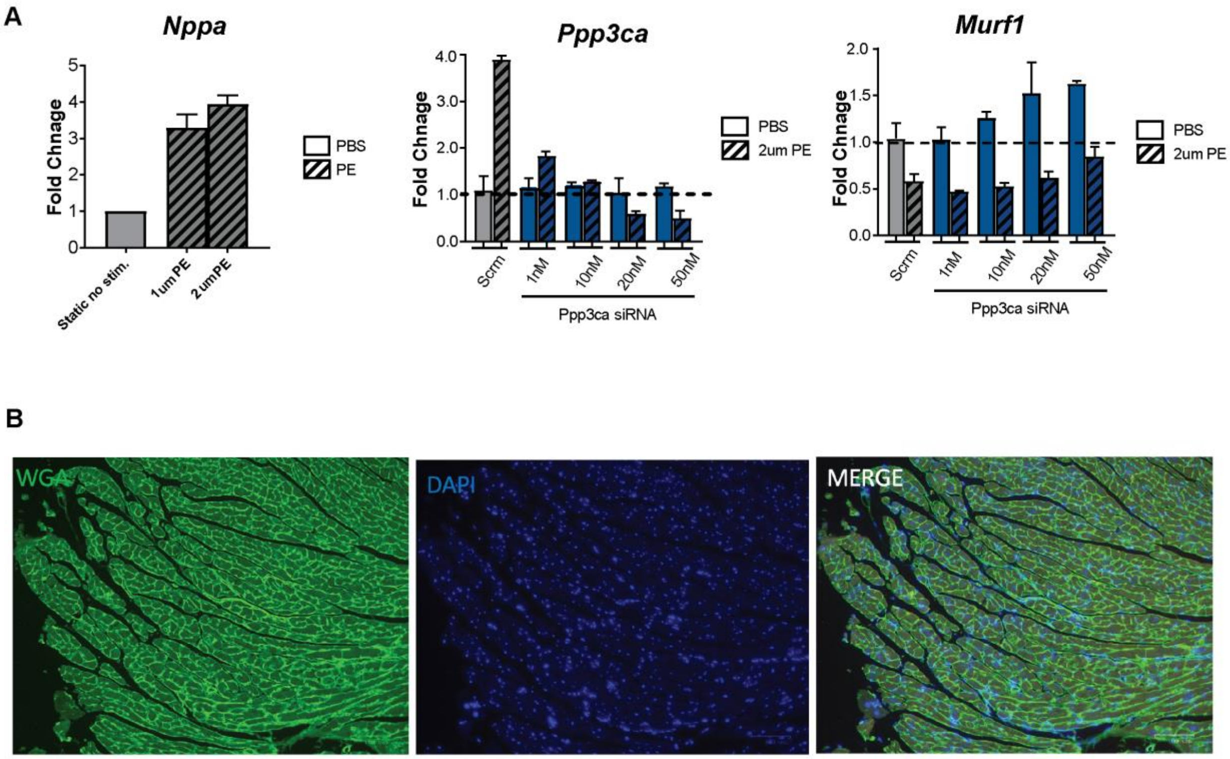

Figure S7. A. Optimal stimulation conditions for inducing *Nppa* upregulation, *Ppp3ca,* and *Murf1* expression in H9c2 fibers on the organ-on-a-chip model administered with different doses of commercial Ppp3ca siRNA (CaN) upon 2 µM PE treatment, evaluated using qRT-PCR. Data is represented as fold change vs its respective control as mean (SD) and derived from experiments with n=3 **B.** Positive immunofluorescence staining of Wheat Germ Agglutinin (WGA, Green) and DAPi nuclear staining (Blue) on heart tissue.

Supplemental Video Material

Video S1. Representative video showing successful transfection of *Cond*-siRNA (red) in beating NRVMs at scale 100 μm, one week after transfection.
